# Lysosomal Targeting with Stable and Sensitive Fluorescent Probes (Superior LysoProbes): Applications for Lysosome Labeling and Tracking during Apoptosis

**DOI:** 10.1038/srep09004

**Published:** 2015-03-11

**Authors:** Xin Chen, Yue Bi, Tianyang Wang, Pengfei Li, Xin Yan, Shanshan Hou, Catherine E. Bammert, Jingfang Ju, K. Michael Gibson, William J. Pavan, Lanrong Bi

**Affiliations:** 1 Department of Chemistry, Michigan Technological University, Houghton, MI 49931; 2 Second Hospital of HeBei Medical University, Shijiazhuang, China 050000; 3 Translational Research Laborator, Stony Brook Mediciney, Stony Brook, NY 11794; 4 Experimental and Systems pharmacology, College of Pharmacy, Washington State University, Spokane, WA 99202; 5 National Human Genome Research Institute, NIH, Bethesda, Maryland 20892

## Abstract

Intracellular pH plays an important role in the response to cancer invasion. We have designed and synthesized a series of new fluorescent probes (Superior LysoProbes) with the capacity to label acidic organelles and monitor lysosomal pH. Unlike commercially available fluorescent dyes, Superior LysoProbes are lysosome-specific and are highly stable. The use of Superior LysoProbes facilitates the direct visualization of the lysosomal response to lobaplatin elicited in human chloangiocarcinoma (CCA) RBE cells, using confocal laser scanning microscopy. Additionally, we have characterized the role of lysosomes in autophagy, the correlation between lysosome function and microtubule strength, and the alteration of lysosomal morphology during apoptosis. Our findings indicate that Superior LysoProbes offer numerous advantages over previous reagents to examine the intracellular activities of lysosomes.

Lysosomes are membrane bound organelles that contain digestive enzymes functioning to recycle damaged organelles as well as digest nucleic acids, polysaccharides, fats and proteins. In the maintenance of cellular homeostasis, lysosomes are active in intracellular signaling, energy metabolism, secretions, and plasma membrane repair. Dysfunction of lysosomal structure or function is associated with multiple pathologies, including inflammation, cancer, neurodegenerative disease, and specific lysosomal storage diseases such as Tay-Sachs^1^,^2^,^3^,^4^,^5^,^6^,^7^,^8^,^9^,^10^,^11^,^12^,^13^,^14^,^15^,^16^. Currently, there are limited numbers of fluorescent probes that efficiently enable a comprehensive evaluation of the structure/function correlates of lysosomes^17^,^18^,^19^,^20^,^21^,^22^,^23^,^24^,^25^,^26^,^27^,^28^,^29^,^30^,^31^,^32^. Cellular compartments with a low internal pH (pH 4.0-6.0), including the lysosome, enable basic amines of low ionic strength to selectively target and thereby explore the synthesis and actions of lysosomes. Neutral red and acridine orange are commonly used to stain acidic organelles such as lysosomes, yet they lack specificity. Conversely, LysoTracker represents a commercially available fluorescent acidotropic probe for lysosome labeling. However, when LysoTracker probes accumulate intracellularly for prolonged periods, the intracellular pH increases which may result in enhanced quenching of the fluorescent dye. Most commercially available lysosome probes require a short excitation wavelength, which considerably restricts the use of these probes in tissue imaging associated with low penetration depth, decreased solubility, and poor photostability due to wavelength restrictions^17^,^18^,^19^,^20^,^21^,^22^,^23^,^24^,^25^,^26^,^27^,^28^,^29^,^30^,^31^,^32^. To address this problem, Belfield et al recently developed a novel, two-photon absorbing fluorescence derivatives exhibiting selectivity for the lysosomes of HCT 116 colon cancer cells^26^. In the current report, we developed alternative lysosome-specific probes that manifest advantages for short- and long-term studies of lysosome structure and function.

## Results and Discussion

### Design Rationale

**Figure 1:**
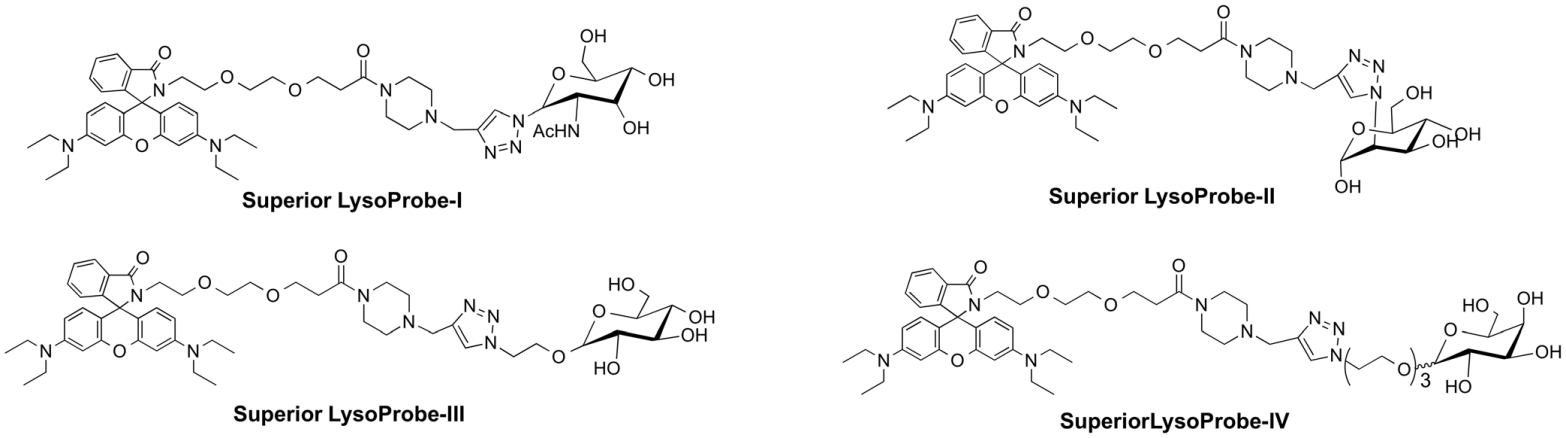
Chemical structures of Superior LysoProbes (I–IV).

In earlier work, we developed several fluorescent probes that aggregate in lysosomes, and they were employed to monitor intracellular pH and localize lysosomes in cultured cells^28^. Nonetheless, these acidotropic probes are comparable to LysoTrackers in that they label compartments based upon their pKa values, thereby decreasing their specificity for lysosomes. To enhance lysosome-targeting specificity, we synthesized a series of novel Superior LysoProbes. We have taken advantage of the fact that lysosomal membrane proteins are heavily glycosylated with numerous N-linked glycans. The latter contain mannose, fucose, galactose, N-acetyl-glucosamine, and sialic acid monosaccharides, which protect lysosomal membrane proteins from degradation by lysosomal proteases such as cathepsins. We hypothesized that conjugation of the parent fluorophore with an N-linked glycan conjugate would aid targeting of fluorescent probes to lysosomes. To examine this hypothesis, we have synthesized a series of new fluorescent probes I–IV (chemical structure shown in Fig. 1) and documented selectivity for lysosomes and the capacity of these probes to label living cells at nanomolar concentrations over an extensive time course. For synthesis of Superior LysoProbes, various N-linked glycan moieties were introduced via “click” chemistry^29^ (Schemes S1 & S2). The spirocyclic structures of the rhodamine lactam-type derivatives were confirmed by NMR. When the spirocyclic compounds exist in the lactam (cyclic amide) form, they lack measurable absorbance and fluorescence in the visible spectrum, yet this returns when converted to the amide form. The binding activity and spirocyclic structure of Superior LysoProbes was confirmed using UV-Vis titration.

### Spectral Properties of Superior LysoProbes I–IV

Superior LysoProbes I–IV were non-fluorescent under basic conditions, whereas a shift to acidic conditions yielded a pink chromophore with strong fluorescence. This indicates that these probes may serve as ‘naked-eye' indicators for [H^+^]. As shown in Figure S1., the fluorescence peak of Superior LysoProbe I–IV was 584 ± 5 nm with minimal intensity beyond pH 7.0. Conversely, fluorescence intensity increased >900-fold as the pH decreased from 7.0 to 4.8. We subsequently examined the standard fluorescence pH titration curve employing 0.07 M buffered solution (0.2 M K_2_HPO_4_–0.1 M citric acid buffer). As showed in Fig. S1, the fluorescence emission at 584 ± 5 nm fluctuated with the pH titration curve of the Superior LysoProbes in buffered potassium phosphate-citric acid. The pH response of Superior LysoProbes I–IV to H^+^ was rapid and its fluorescence intensity saturation was reached within a few minutes at the range of pH 4.80 ~ 6.00, an important advantage for real-time monitoring of lysosomal pH changes. Additionally, Superior LysoProbes maintain extensive fluorescence and high concentration at low pH, offering distinct advantages over other fluorescent probes.

### Intracellular localization

**Figure 2:**
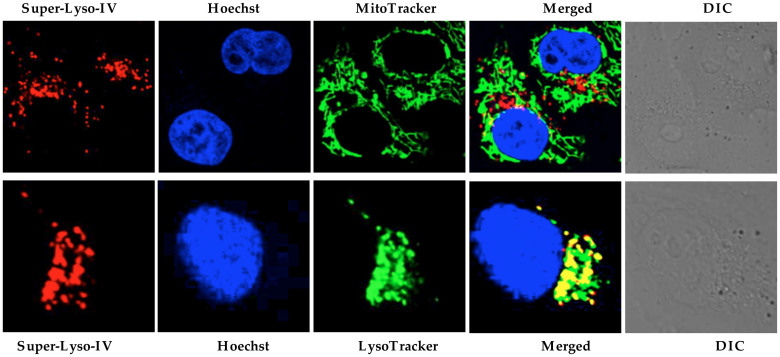
Intracellular distribution of Superior LysoProbe (IV) (1 μM) compared to MitoTracker (80 nM) and LysoTracker Green (2 μM). HeLa cells were imaged on an inverted laser scanning fluorescent microscope (Olympus) using a 60 × oil immersion objective lens.

To examine the intracellular localization of the synthesized probes, double staining with MitoTracker in HeLa cells was performed. Separate labeling patterns, and a lack of co-localization of the probe with mitochondria, were observed after 30 min incubation (Fig. 2 and Fig. S2–S4). To further explore labeling patterns, HeLa cells were stained with LysoTracker Green (a commercial lysosome selective stain), which revealed colocalization of Superior LysoProbe IV with lysosomes (Fig. 2). Hoechst 33342 was employed for nucleus staining, in order to confirm cell viability throughout the experiment. To estimate the degree of colocalization of Superior LysoProbes and LysoTracker, a quantitative analysis of confocal images was performed. The Pearson's correlation coefficient (POC) and the Mander's overlap coefficient (MOC) were used to quantify the degree of co-localization between fluorophores. The Pearson's correlation coefficients for Superior LysoProbe I–IV and LysoTracker were high. Additionally, the overlap coefficients of the two fluorescence patterns were also relatively high (Table S1).

**Figure 3:**
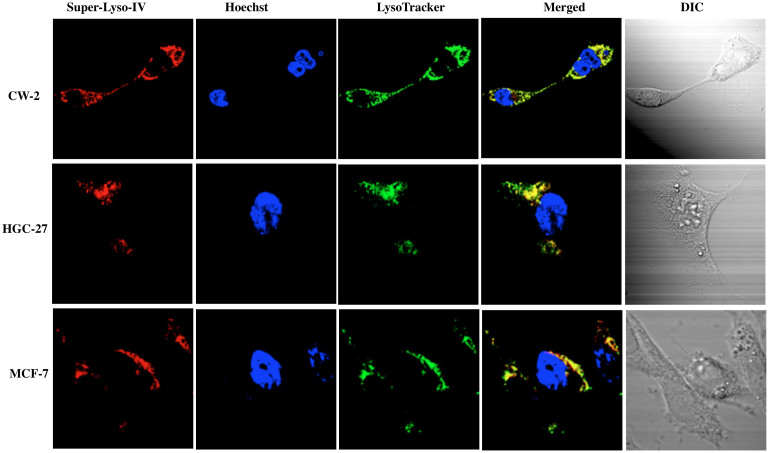
The fluorescence intracellular localization of Superior LysoProbe (IV) (1 μM) was examined in an additional three cancer cell lines: gastric cancer-derived HGC-27 cells, human colon cancer-derived CW-2 cells, and human breast cancer MCF-7 cell lines. The intracellular distribution of Superior LysoProbe (IV) (1 μM) compared to LysoTracker Green (2 μM) in human colon cancer-derived CW-2 cells, gastric cancer-derived HGC-27 cells, and human breast cancer MCF-7 cell lines is shown. Superior LysoProbe (IV) (1 μM) was incubated with cells in non-FBS DMEM media for 15 min., and then counterstained with LysoTracker (2 μM, weak green fluorescence), Hoechst 33342 (1 μg/mL, blue). The cells were imaged on a confocal laser-scanning fluorescent microscope (Olympus) using a 60 × oil immersion objective lens.

The fluorescence intracellular localization of Superior LysoProbes was further examined in three additional cancer cell lines: gastric cancer-derived HGC-27 cells, human colon cancer-derived CW-2 cells, and human breast cancer MCF-7 cells. As shown in Fig. 3, the fluorescence patterns of Superior LysoProbe IV and LysoTracker overlapped exactly, indicating lysosomal localization for Superior LysoProbe IV, and confirming that Superior LysoProbes localized in lysosomes in different cancer cell lines.

### Lysosome-targetable moiety

**Figure 4:**
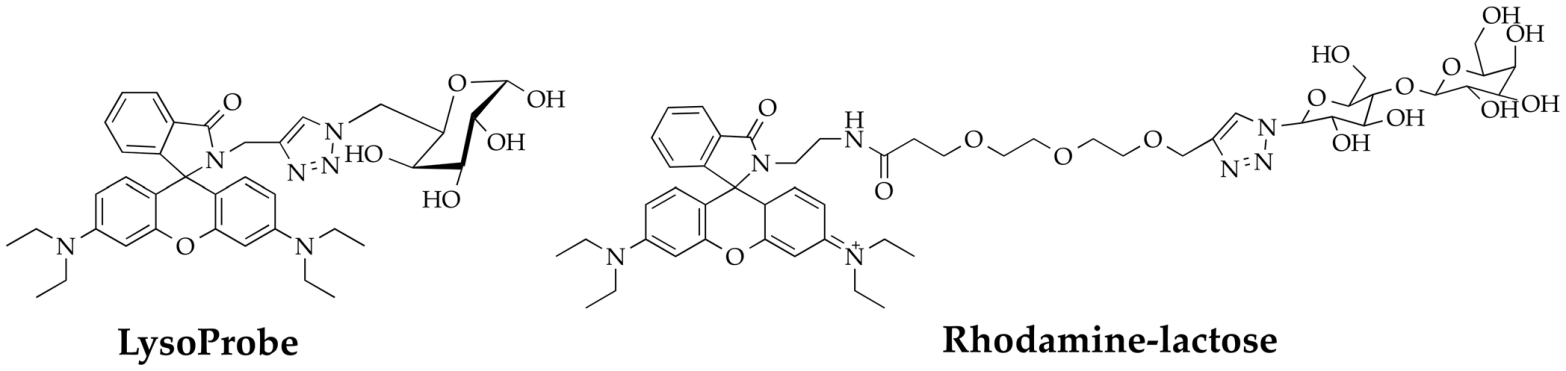
Chemical structures of two control compounds: rhodamine-lactose conjugate and a lysosome-specific probe-LysoProbe (without PEG linker).

**Figure 5:**
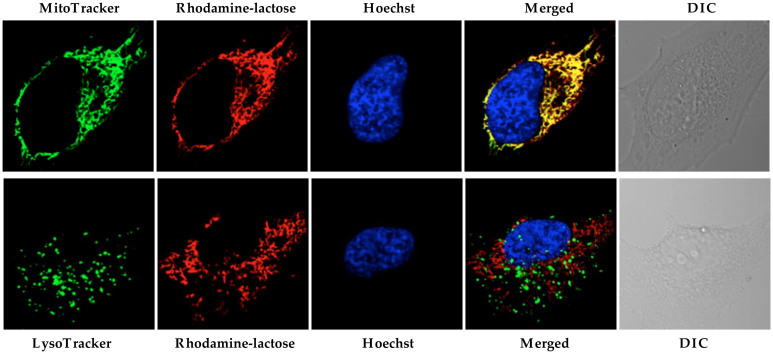
Intracellular distribution of a control compound, rhodamine-lactose (1 μM) compared to MitoTracker (80 nM) and LysoTracker Green (2 μM). HeLa cells were imaged on an inverted laser scanning fluorescent microscope (Olympus) using a 60 × oil immersion objective lens.

We hypothesized that the N-linked glycan moiety of Superior LysoProbes would enhance lysosomal targeting. The fluorescence of Superior LysoProbes I–IV was distributed in a punctate pattern only in lysosomes rather than diffusely throughout the cell. Conversely, the fluorescent images of the precursor of Superior LysoProbes (Scheme S1) was non-punctate and diffuse after several hours, suggestive of rapid diffusion leading to reduced probe sensitivity. Thus, the N-linked glycan moieties appear to enhance selective lysosomal aggregation. To further confirm our hypothesis, we synthesized an additional control compound, rhodamine-lactose (lacking N-linked glycan; chemical structure shown in Fig. 4). This derivative did not target to lysosomes. As shown in Fig. 5, rhodamine-lactose and LysoTracker did not co-localize. However, rhodamine-lactose did co-localize with MitoTracker. These results confirmed the requirement for an N-linked glycan moiety for efficient lysosomal-targeting and intracellular retention.

PEGylation has been utilized to modify the pharmacokinetic properties of small molecules through modulation of hydrophobicity. Our results indicated that the addition of a short PEG moiety to the linker between the lysosome-targeting moiety and the fluorophore moiety increased the cellular uptake of Superior LysoProbes. Moreover, the linker enhanced target-to-background fluorescence ratios when compared our first generation LysoProbes (which lacked PEG linkers; chemical structure shown in Fig. 4)^30^.

### Intracellular retention

**Figure 6:**
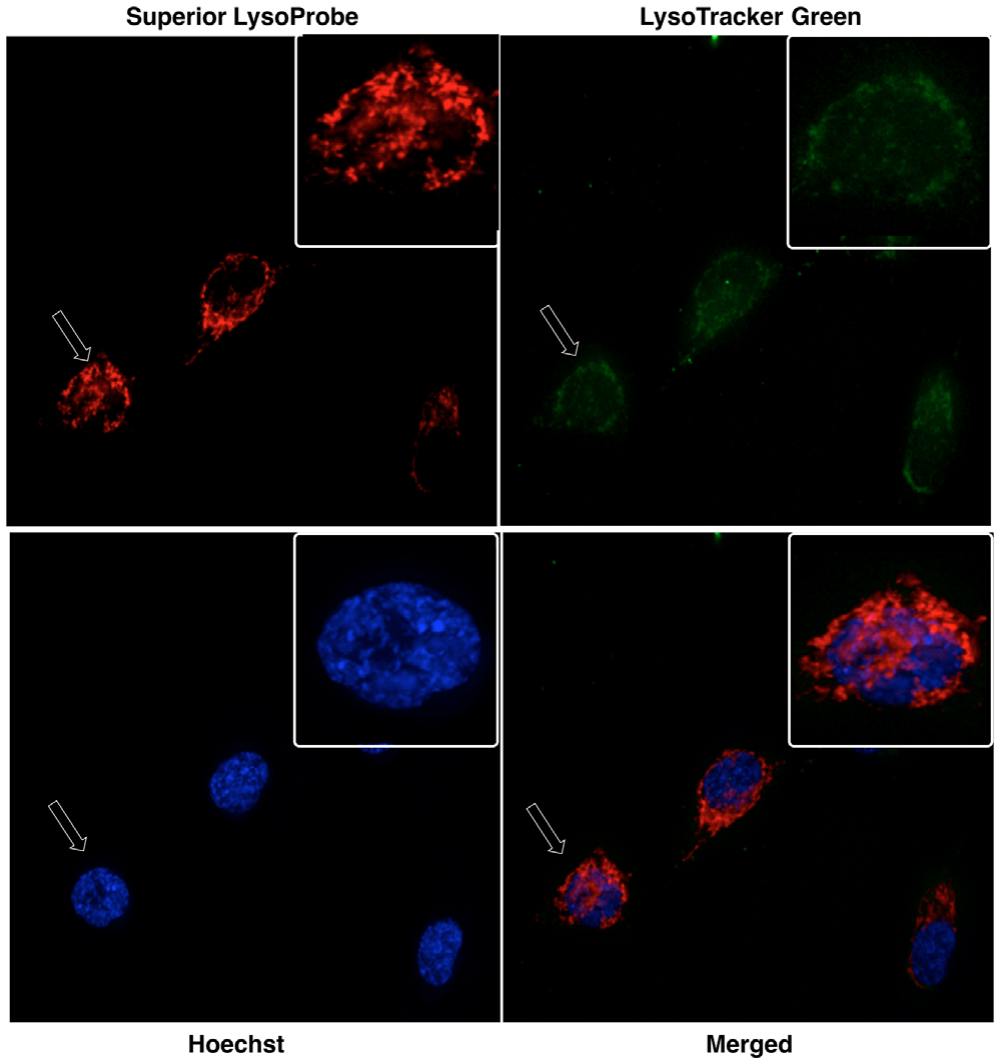
Confocal laser-scanning fluorescent images of Superior LysoProbe (IV) in HeLa cells. Superior LysoProbe IV (1 μM, red) was incubated with cells in non-FBS DMEM media for 15 min., and then counterstained with LysoTracker (2 μM, weak green fluorescence), Hoechst 33342 (1 μg/mL, blue), and followed by 48 h incubation. All images were acquired using a 60 × oil immersion objective lens.

We performed long-term retention studies following cell loading with fluorescent probes to assess the extent of intracellular retention. For these studies, Superior LysoProbe and LysoTracker Green were incubated with HeLa cells for 48 h and their fluorescent changes evaluated. Within 2 hours, HeLa cells loaded with LysoTracker Green displayed no observable fluorescence. Conversely, there was still significant fluorescence in HeLa cells loaded with Superior LysoProbe at 48 h, and the intracellular staining pattern remained punctate (images shown in Fig. 6), suggesting significant probe retention in the lysosome.

### Intracellular pH measurement

**Figure 7:**
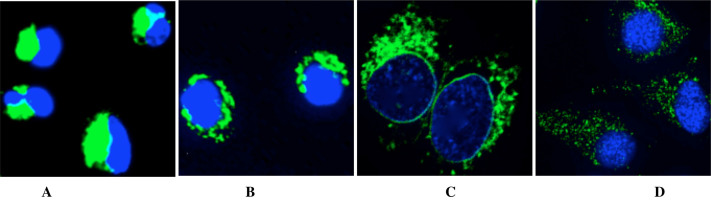
Intracellular pH measurements using Superior LysoProbe (IV) (1 μM) in ARPE cells: After washing three times with corresponding pH buffers, cells were further incubated with nigericin and then imaged in buffers maintained at: (A) pH 4.0; (B) pH 4.5; (C) pH 5.0; and (D) pH 7.4. Cells were imaged on an inverted laser scanning fluorescent microscope (Olympus) using a 60 × oil immersion objective lens.

We next investigated alterations in regional pH values by treating ARPE -19 cells (a human retinal pigment epithelial cell line) with nigericin, a H^+^/K^+^ antiporter, and subsequently followed the fluorescence imaging of Superior LysoProbes at various pHs. Intracellular and extracellular pH was maintained in equilibrium throughout the experiment. The intracellular pH-responsiveness to Superior LysoProbes was inversely correlated as a function of pH (e.g., lower pH, higher intracellular probe accumulation (Fig. 7)). Conversely, ARPE-19 cells maintained at an acidic extracellular pH demonstrated enhanced dispersion of lysosomes that were located proximal to the periphery of the cell (Fig. 7A–C). On the other hand, lysosomes in cells maintained at a pH approximating neutrality (7.4) revealed lysosomes that were primarily localized to the perinuclear region (Fig. 7D).

### Real-time Imaging of dysfunctional lysosomes during apoptosis induced by lobaplatin

Lobaplatin, a novel cisplatin analogue, has shown a promising anticancer profile in multiple carcinomas through inhibition of cell proliferation^31^. Here, we investigated the cholangiocarcinoma (CCA) cell line RBE, since there is no detailed information available on the effects of lobaplatin on human CCA. We were particularly interested in assessing the role of lysosomes during lobaplatin-induced apoptosis.

#### Lobaplatin-associated arrest of cell cycle progression in the G0/G1 phase

**Figure 8:**
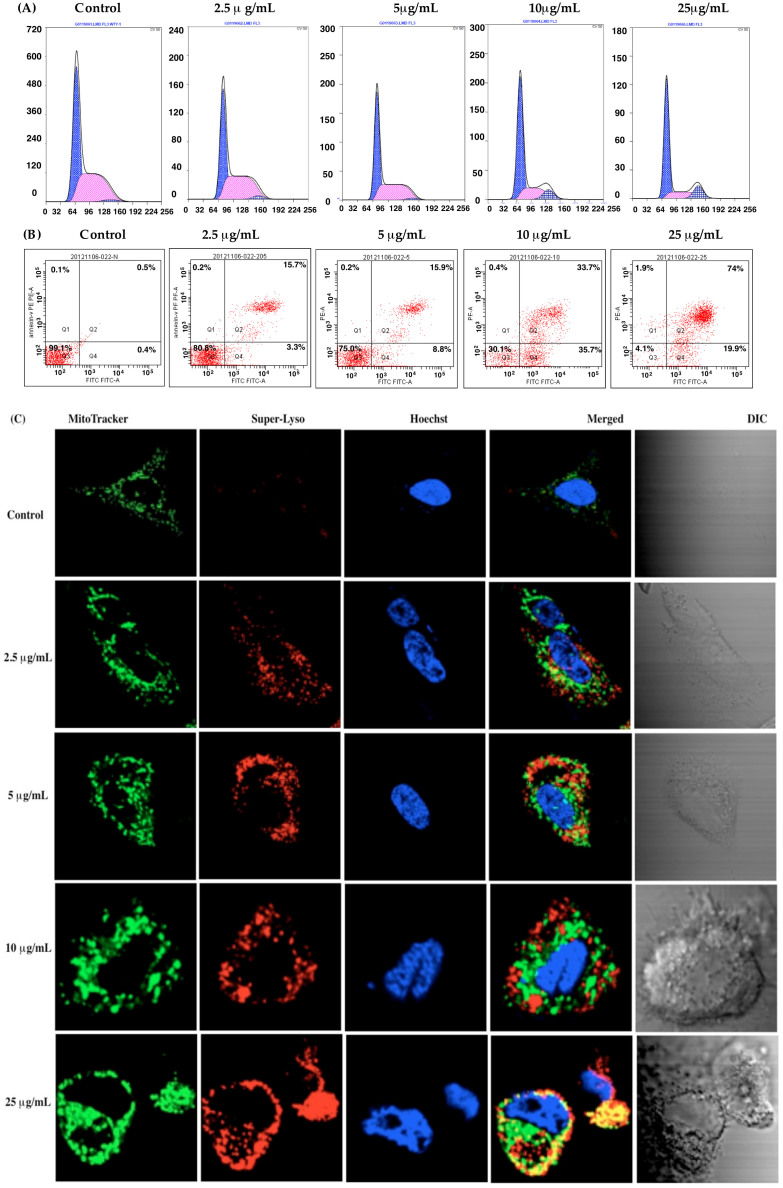
Effect of lobaplatin treatment in human cholangiocarcinoma RBE cells. (A) RBE cells were exposed to various concentrations of lobaplatin for 24 h followed by analysis of the cell cycle via flow cytometry; (B) RBE cells were exposed to various concentrations of lobaplatin for 24 h and followed by flow cytometry analysis using the Annexin V-FITC apoptosis detection kit; (C) Confocal fluorescent laser scanning images showing mitochondrial and lysosomal changes in RBE cells following treatment with lobaplatin (2.5 ~ 25 μg/mL). RBE cells were counterstained with Superior LysoProbe (IV) (1 μM, red), Hoechst 33342 (1 μg/mL) and MitoTracker (80 nM). Cells were imaged on an inverted laser scanning fluorescent microscope (Olympus) using a 60 × oil immersion objective lens.

Figure S5 displays the time- and concentration-dependent inhibition of cell growth following lobaplatin treatment. The proliferation of RBE cells was reduced >50% with 5 to 25 μg/mL lobaplatin (48 h incubation), with further growth reductions at 72 h incubation. Lobaplatin treatment induced circularization of RBE cells that are normally spindle-shaped. Fluorescence-activated cell sorting (FACS) analysis was then performed following propridium iodide (PI) staining to assess the cell cycle distribution of lobaplatin-treated cells. Upon addition of lobaplatin, RBE cells began to accumulate in the G0/G1 phase (24 h incubation), and the S and G2/M phase cell populations were reduced. Because of this, the cells lacked the ability to undergo cell division, resulting in an eventual arrest of the RBE cell cycle. Progression to cell division ceased (Fig. 8A), and we found a close correlation between lobaplatin dose and percentage of cells arrested at the G1/S phase of the cell cycle.

#### Lobaplatin-induced apoptosis

For this study, RBE cells were treated with lobaplatin for 24 h, stained and analyzed. The percentage of apoptotic cells determined using the Annexin V-FITC/PI apoptosis detection kit, is displayed in Fig. 8B. At lobaplatin concentrations > 10 μg/ml, cells demonstrated dose-dependent enhancement of apoptosis in comparison to untreated cells. Since an additional mechanism to suppress cell growth is cell cycle arrest, the well-described capacity of anticancer drugs such as cisplatin and oxaliplatin to arrest cell cycle progression may represent an additional explanation for the observed cytotoxicity of lobaplatin.

### Lobaplatin-induced organelle dysfunction

Previous studies suggest that platinum-based anticancer drug-induced cell apoptosis involves mitochondria-dependent pathways of cell death^33^,^34^,^35^. Alternative cell death pathways may involve lysosomal membrane permeabilization and/or autophagy^36^,^37^,^38^,^39^. Recent studies have shown that lobaplatin induces apoptotic cell death, but the underlying molecular mechanisms remain to be elucidated. The involvement of mitochondria and lysosomes in lobaplatin-induced apoptosis were further characterized in our studies. To monitor organelle changes during apoptosis induced by lobaplatin, we stained RBE cells with Superior LysoProbe (lysosome), Hoechst 33342 (nuclei) and Mitotracker (mitochondria) (Fig. 8C).

Healthy mitochondria exist as a dynamic network controlled by fusion and fission events and punctate structures in the perinuclear region, with an overall filamentous morphology. In untreated cells, mitochondria were equally dispersed throughout the cell with an appropriate morphology and filamentous network. Conversely, low concentrations of lobaplatin induced discontinuity of mitochondrial networks in a concentration-dependent manner. Treatment of cells with higher lobaplatin concentrations (>10 μg/mL) resulted in further fragmentation of mitochondrial filament networks. Further increases in lobaplatin resulted in mitochondrial localizalization to the perinuclear region, and the organelles assumed a swollen, fragmented morphology. For our assessments, we captured images in random fields and scored the cell number by identification of a filamentous and fragmented mitochondrial structure. Cells treated with lobaplatin (2.5 μg/mL) revealed approximately 16% cells with fragmented mitochondrial morphology, whereas mitochondria in cells treated with lobaplatin at >10 μg/mL demonstrated 85% of cells with this identical fragmented morphology.

To gauge the role of lysosomes in apoptosis, we labeled lysosomes with Superior LysoProbe and monitored morphological alterations associated with lobaplatin. While control cells displayed a punctate localization in the perinuclear region, lobaplatin-treated lysosomes swelled and accumulated in the cytosol with increasing lobaplatin administration (Fig. 8C). At concentrations beyond 10 μg/ml, lobaplatin induced alterations in nuclear morphology (Fig. 8C). Further, treatment with lobaplatin produced a gradual and concentration-dependent increase in Superior LysoProbe fluorescence intensity, suggesting that lobaplatin induced alterations of lysosomal pH (Fig. 8C). The latter changes correlated with early apoptosis, suggesting that lysosomal perturbations were involved in the onset of apoptosis. Interestingly, we found a positive correlation between the mean cytotoxicity index and mitochondrial fragmentation, and the evidence suggested that lobaplatin also induced mitochondrial dysfunction. These data suggested that prolonged exposure to cytotoxic concentrations of lobaplatin were associated with permeabilization of lysosomal membranes and ensuing mitochondrial dysfunction. All of the preceding results indicate that the sensitivity of RBE cells to lobaplatin can be effectively gauged through evaluation of lysosome-associated parameters determined using Superior LysoProbe. Our studies further suggest that lobaplatin-induced cell apoptosis may involve both mitochondrial damage and lysosomal membrane destabilization. The predicted coordination between mitochondria and lysosomes during apoptosis requires further investigation (Fig. S7).

#### Chloroquine (CQ) sensitizes RBE cells to lobaplatin treatment

LC3 (Microtubule-associated protein 1A/1B-light chain 3) is a soluble protein that is universally distributed in cultured cells. During autophagy, autophagosomes engulf cytosolic proteins and organelles. Autophagosomes fuse with lysosomes to form autophagolysosomes, and intra-autophagosomal components are degraded by lysosomal hydrolases (Fig. S8)^38^,^39^. Thus, lysosomal turnover of the autophagosomal marker LC3 reflects starvation-induced autophagic activity, and detecting LC3 by immunofluorescence is a reliable method for monitoring autophagy and autophagy-related processes, including autophagic cell death. Chloroquine (CQ) is a synthetic 4-aminoquinoline that has been used for the treatment of malaria. CQ functions as a weak base and can be trapped in acidic cellular compartments including lysosomes. Previous studies suggested that CQ inhibits lysosomal acidification and prevents autophagy through blockade of autophagosome fusion and degradation, a feature of CQ exploited in the current study.

**Figure 9:**
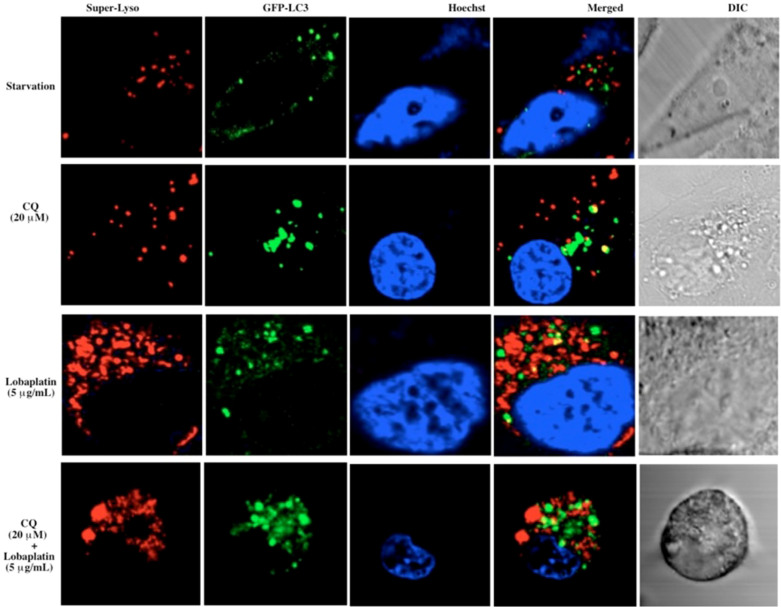
Confocal laser-scanning fluorescent images of RBE cells treated with lobaplatin (5 μg/mL) with and without CQ (20 μM) for 24 h. GFP-LC3 (green fluorescence) transfected RBE cells were labeled with Superior LysoProbe (IV) (1 μM, red fluorescence), and Hoechst 33342 (1 μg/ml, blue fluorescence). Cells were imaged on an inverted laser scanning fluorescent microscope (Olympus) using a 60 × oil immersion objective lens.

Autophagy may represent a cell-survival pathway in chemotherapy. To assess parameters of autophagy, RBE cells were transfected with the autophagosome marker LC3^38^,^39^ To monitor lysosomes, the GFP-LC3 transfected RBE cells were labeled with Superior LysoProbe IV (1 μM). Untreated cells demonstrated a primarily diffuse cytoplasmic staining of GFP-LC3 with very few punctuate autophagosomes and lysosomes (images not shown). An induction of autophagosome formation was observed in RBE cells following autophagic stimuli (starvation in serum-free medium) (Fig. 9, row 1). In contrast, treatment of RBE cells with CQ (20 μM, 24 h) resulted in a massive induction of GFP-LC3-positive autophagosomes. Further, CQ-treated cells displayed a marked increase in the number and size of lysosomes (Fig. 9, row 2), which retained Superior-LysoProbe fluorescence, indicating that the lysosomes did not lose their internal acidic nature. Yellow regions in the merged images indicate autophagolysosome formation, a result of combining GFP-LC3 (green fluorescence) and Superior LysoProbe (red fluorescence) colocalization. After lobaplatin treatment alone (5 μg/mL, 24 hr), a significant increase in the percentage of cells that were highly labeled with Superior LysoProbe (Fig. 9, row 3) was observed as compared to control untreated cells, suggesting that lobaplatin treatment alone resulted in an increase in lysosomal activity. However, after the combinatorial treatment with CQ (20 μM) + lobaplatin (5 μg/mL), many vesicles retained GFP staining and Superior-LysoProbe fluorescence, but an absence of yellow combinatorial fluorescence was revealed in the merged image (Fig. 9, row 4), indicating that combined treatment induced a marked accumulation of vesicles that included autophagosomes and lysosomes, but a blockade of the fusion of autophagosomes with lysosomes. We speculate that this result was the result of lysosomal dysfunctional. It has been reported elsewhere that CQ blocks autophagy via inhibition of lysosomal proteases and autophagosome-lysosomal fusion, consistent with our results suggesting that sensitization to lobaplatin treatment was induced by CQ treatment.

#### Lobaplatin-induced cytoskeleton alterations in RBE cells

**Figure 10:**
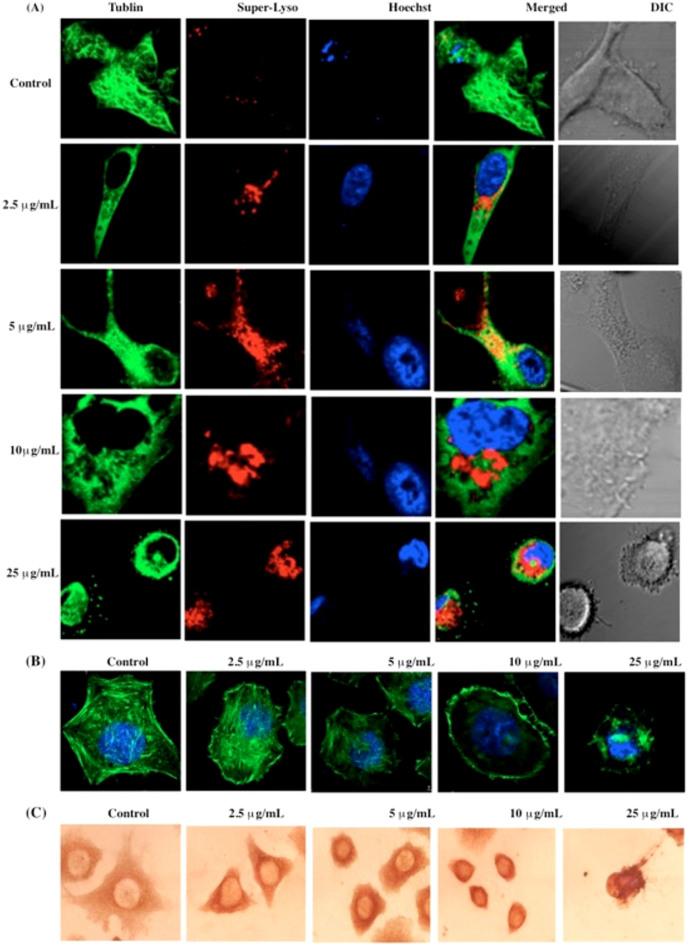
(A) Detection of extensive microtubule disruption and lysosome perturbation in RBE cells exposed to lobaplatin (2.5 ~ 25 μg/mL). RBE cells were labeled with Tublin-GFP (green fluorescence), Superior LysoProbe (IV) (10 μM, red fluorescence) and Hoechst 33342 (1 μg/ml, blue fluorescence); (B) The dissolution of cytoskeletal F-actin structural characteristics was observed in RBE cells exposed to lobaplatin (2.5 ~ 25 μg/mL). F-actin exhibits green fluorescence and nuclei exhibit blue fluorescence associated with Hoechst 33342 staining. RBE cells were imaged on an inverted laser scanning fluorescent microscope (Olympus) using a 60 × oil immersion objective; (C) immunohistochemical images of α-Tublin in RBE cells exposed to lobaplatin (2.5 ~ 25 μg/mL).

The previous findings of translocated organelles suggested that the cellular cytoskeleton might play a role in lobaplatin-induced apoptosis. To examine this possibility, we next investigated the relationship(s) between lysosomes and the cytoskeleton, since it was reasonable to assume that lysosomal relocation might be correlated with cytoskeleton integrity. Moreover, it is probable that lysosomal intracellular distribution would correlate with the extent of microtubule extension into the cytoplasm. In our studies evaluating these parameters, control cells demonstrated visible lysosomes distributed in the cell periphery superimposed on the microtubule (Fig. 10). Similar results were found at low concentrations of lobaplatin (2.5 μg/mL), but at higher lobaplatin concentrations the microtubules were interrupted and condensed to the perinuclear region. At these concentrations of lobaplatin, the lysosomes aggregated near the nucleus where the microtubule-organizing center was disrupted (Fig. 10A). Moreover, we found that the absolute levels of cytoskeletal F-actin protein was significantly diminished in RBE cells exposed to lobaplatin (2.5 ~ 25 μg/mL) (Fig. 10B), a finding further confirmed by immune-histochemical assays of α-tubulin in RBE cells exposed to lobaplatin (2.5 ~ 25 μg/mL) (Fig. 10C). Although we suspect that the translocation of lysosomes may occur along microtubules, the present assays only indirectly suggest an association between lysosomes and cytoskeletal elements, a process that is under further investigation in our laboratory.

## Conclusion

In the current study, the use of Superior LysoProbes enabled us to visualize the role of lysosomes in autophagy, the correlation between lysosome activity and microtubule strength, and the lysosomal morphological changes during apoptosis in human cholangiocarnioma RBE cells induced with lobaplatin. Our findings suggest that an absence of cytotoxicity, high cellular permeability, long-lived intracellular fluorescence, and selective accumulation within lysosomes make Superior LysoProbes preferable to commercially available lysosome probes. Furthermore, our pilot data suggest that investigation of the lysosome-associated parameters may be useful in predicting the sensitivity of cancer cells to chemotherapy. Moreover, the data presented herein suggest that disruption of lysosomal function by CQ may provide a potentially useful therapeutic intervention in certain cancer settings. The increased sensitivity of cancer cells to CQ treatment would provide a natural therapeutic index, yet the challenge will remain as to how to optimize this approach in both chemoprevention and cancer treatment.

## Methods

### Synthesis of Superior LysoProbes (I–IV)

Synthesis schemes, experimental details of Superior Lysoprobes and fluorescent images are provided as supplementary information.

### Cell Culture

HeLa cells (CCL-2) were obtained from American Type Cell Culture collection (ATCC) and grown in Eagle's Minimal Essential Medium (EMEM) and 10% FBS (Sigma-Aldrich, heat inactivated) with incubation at 37°C in 5% CO_2_. One or two days prior to imaging, the cells were passaged and plated in phenol red-free medium on 4-well chamber slides (Corning, Corning NY), and allowed to grow to 50–70% confluence. The retinal pigment epithelial cell line ARPE-19 was obtained from ATCC and grown in DMEM/Ham's F12 1:1 (Hyclone, Fisher Sci.) containing 10% FBS (Sigma-Aldrich, heat inactivated). ARPE-19 (50.000 cells/well) cells were grown in 24-well plates for 12 h. RBE cells were obtained from ATCC and cells were maintained in RPMI-1640 (Gibco BRL, Carlsbad, CA, USA) supplemented with 10% fetal bovine serum (Gibco) and 100 U/mL penicillin and 0.1 mg/mL streptomycin (Penicillin and Streptomycin Solution (100X), Gibco, USA) at 37°C in a humidified atmosphere containing 5% CO_2_. CW-2 cells were obtained from ATCC and cultured in Dulbecco's modified Eagle's medium supplemented with 10% heat-inactivated fetal bovine serum, penicillin (final concentration, 100 U/ml), and streptomycin (final concentration, 0.1 mg/ml), in a humidified atmosphere of 5% CO_2_ and 95% air at 37°C. HGC-27 cells were obtained from ATCC and maintained in RPMI 1640 medium containing 10% FBS, 100 U/mL penicillin, and 100 U/mL streptomycin at 37°C with 5% CO_2_. Cells were passaged at 80% confluency using 1 mmol/L ethylene diamine tetraacetic acid (EDTA)-0.025% trypsin. The MCF-7 cell line was obtained from ATCC and maintained as an attached monolayer culture in commercially defined RPMI 1640 medium, supplemented with 10% (v/v) heat-inactivated fetal bovine serum (FBS), 2 mM L-glutamine, 100 U/mL and 100 μg/mL penicillin–streptomycin, and 25 μM 4-(2-hydroxyethyl)-1-piperazineethanesulfonic acid (HEPES).

### Cell staining procedures and Co-localization studies

Cells were adjusted to a density of 50000 per cm^2^, incubated for 48 h, and then washed with cell culture medium twice. For the Superior LysoProbe and LysoTracker co-stain, a solution of Superior LysoProbe I–IV (1 μM) and LysoTracker Green (2 μM) in cell culture medium was added to pre-washed cells and incubated at 37°C for 45 min. For the Superior LysoProbe I–IV and MitoTracker co-stain, a solution of Superior LysoProbe I–IV (1 μM) and MitoTracker (80 nM) in cell culture medium was added to the pre-washed cells and incubated at 37°C for 30 min. To achieve nuclear staining, cells were incubated with 1 μM Hoechst at 37°C for 15 min prior to imaging. Confocal fluorescence imaging studies were performed with an Olympus laser-scanning microscope with a 60× oil-immersion objective lens. Image analysis was performed using Image J (National Institute of Health). For quantitative analysis of fluorescence of the confocal images, the threshold of the images was set to 10, the area of fluorescence was selected with the “create selection” program function, and the fluorescence intensity of the comprehensive image was measured.

### For intracellular pH measurement assay

ARPE-19 cells were treated with 10 μM nigericin and equilibrated for 2 min employing an intracellular pH calibration buffer kit (Life Technologies) prior to obtaining images using previously published procedures.^28^

### Proliferation assay

The cytotoxicity of lobaplatin to RBE cells was examined using a cell proliferation assay. Cells were seeded in a 96-well microtiter plate at 5 × 10^4^ cells/well, and cultured for 24 hours prior to exposure to lobaplatin of varying concentrations for 48 hours. 10 μL of a CCK-8 solution was added to each well of the plate followed by incubation for 2 hours. The absorbance was quantified at optical density (OD) 450 nm using a SpectraMax 190 Microplate Reader (Molecular Devices, Sunnyvale, CA, USA). Six wells were used for each concentration, and the mean and SD determined. The inhibition of cell proliferation was calculated via application of the following formula: inhibition rate (IR) = [1- (OD treated/OD control) × 100%]. The concentration to achieve 50% inhibition (IC_50_) was calculated by nonlinear regression using the mean data values obtained in triplicate independent experiments. Cell morphologic changes were assessed using a phase-contrast microscope (CKX41, Olympus. Japan) following a 24 hours incubation with lobaplatin.

### Cell cycle distribution analysis

The effect of lobaplatin on RBE cell cycle distribution was determined by FACS analysis. Cells were exposed to different concentrations of lobaplatin for 24 hours, and then harvested, washed twice with PBS and fixed with 70% ethanol solution at 4°C overnight. Following centrifugation, cell pellets were stained with 5 μg/mL PI and 1 μg/mL RNase A at 4°C for 30 minutes. The samples were analyzed by FACScan flow cytometer with CellQuest software. The distribution of cells in various stages of the cell cycle was determined using ModFit LT software.

### Cell apoptosis analysis

Cell apoptosis was evaluated using the Annexin V-FITC Apoptosis Detection Kit (BioVision, CA, USA.). RBE cells were exposed to various concentrations of lobaplatin (0–25 μg/mL) for 24 hours and then cells were harvested and re-suspended in 1 × Binding Buffer. 5 μL of Annexin V-FITC and 5 μL of propidium iodide (PI) were added to RBE cells and the cells subsequently analyzed by flow cytometry.

### Statistical analysis

Statistical calculations were carried out with the SPSS 15 for Windows software package. Results are expressed as the mean ± SD. Student's *t* test was used for statistical analyses and P < 0.05 was chosen as the threshold for significance.

## Author Contributions

L.B. designed the study. X.C. did the synthesis of these fluorescent probes, Y.B., T.W. and P.L. performed the cell assays. S.H. and X.Y. performed the probes characterization. L.B., C.E.B., K.M.G., J.J. and W.J.P. performed data analysis, wrote and edited the manuscript.

## Supplementary Material

Supplementary Information Lysosomal Targeting with Stable and Sensitive Fluorescent Probes (Superior LysoProbes): Applications for Lysosome Labeling and Tracking during Apoptosis
