# Supplementary material for: Lysosomal Targeting with Stable and Sensitive Fluorescent Probes (Superior LysoProbes): Applications for Lysosome Labeling and Tracking during Apoptosis
Source: Sci Rep. 2015 Mar 11;5:9004. doi: 10.1038/srep09004 (PMC4355733; doi:10.1038/srep09004)
Supplement: Supplementary Information — Lysosomal Targeting with Stable and Sensitive Fluorescent Probes (Superior LysoProbes): Applications for Lysosome Labeling and Tracking during Apoptosis [file srep09004-s1.pdf]

Lysosomal Targeting with Stable and Sensitive Fluorescent Probes (Superior LysoProbes): Applications for Lysosome Labeling and Tracking During Apoptosis

Xin Chen,[†‡] Yue Bi,[∫‡] Tianyang Wang,[∫] Pengfei Li,[∫] Xin Yan,[†] Shanshan Hou,[†] Catherine E. Bammert,[†] Jingfang Ju,[*¶] K. Michael Gibson,[*§] William J. Pavan,[⊥] Lanrong Bi[*†]

[†]Department of Chemistry, Michigan Technological University, Houghton, MI 49931

[∫]Second Hospital of HeBei Medical University, Shijiazhuang, China 050000

[¶]Translational Research Laboratory, Stony Brook Medicine, Stony Brook, NY 11794

[§] College of Pharmacy, Washington State University, Spokane, WA 99202

[⊥] National Human Genome Research Institute, NIH, Bethesda, Maryland 20892

*Corresponding authors: lanrong@mtu.edu; mike.gibson@wsu.edu; jingfang.ju@stonybrookmedicine.edu.

[‡]: These authors contributed equally to this work.

Table of Contents:

General Method and Synthesis of Superior LysoProbes I-IV……………………………………………………….2-7
Spectral Properties of Probes…………………………………………………………………………………….8-11
Cell Assays and imaging…………………………………………………………………………………….12-19
Proposed mechanisms of lobaplatin………………………………………………………………………………20
Schematic model of the steps involved in autophagy…………………………………………………………..21
NMR Spectra……………………………………………………………………………………………………..22-33

Experimental Section:

Reagents and solvents available from commercial sources were used as received unless otherwise noted. Thin layer chromatography (TLC) was performed using Sigma-Aldrich TLC plates, silica gel 60F-254 over glass support, 0.25 μm thickness. Flash column chromatography was performed using Alfa Aesar silica gel, particle size 230-400 mesh. Melting points were determined using a MELTEMP® melting point apparatus and were uncorrected. ^{1}H and ^{13}C NMR spectra were measured with a Varian UNITY *INOVA* instrument at 400 MHz and 100 MHz, respectively. The chemical shifts (δ) were reported in reference to solvent peaks (residue $CHCl_3$ at δ 7.24 ppm for ^{1}H and $CDCl_3$ at δ 77.00 ppm for ^{13}C). High-resolution mass spectra (HR-MS) were obtained on a JEOL JMS HX 110A mass spectrometer. UV-vis spectra were recorded on a Perkin Elmer Lambda 35 UV/Vis Spectrometer equipped with PTP 1+1 Peltier Temperature Programmer accessory at 37 °C. Fluorescence spectra were obtained with a Horiba Jobin Yvon Fluoromax-4 spectrofluorometer using an excitation wavelength of 480 nm and slit widths of 5 nm. Fluorescence cells with optical pathlength of 10 × 10 mm were used. Fluroscence imaging was performed on a Zeiss Axiovert 200M with Apotome.

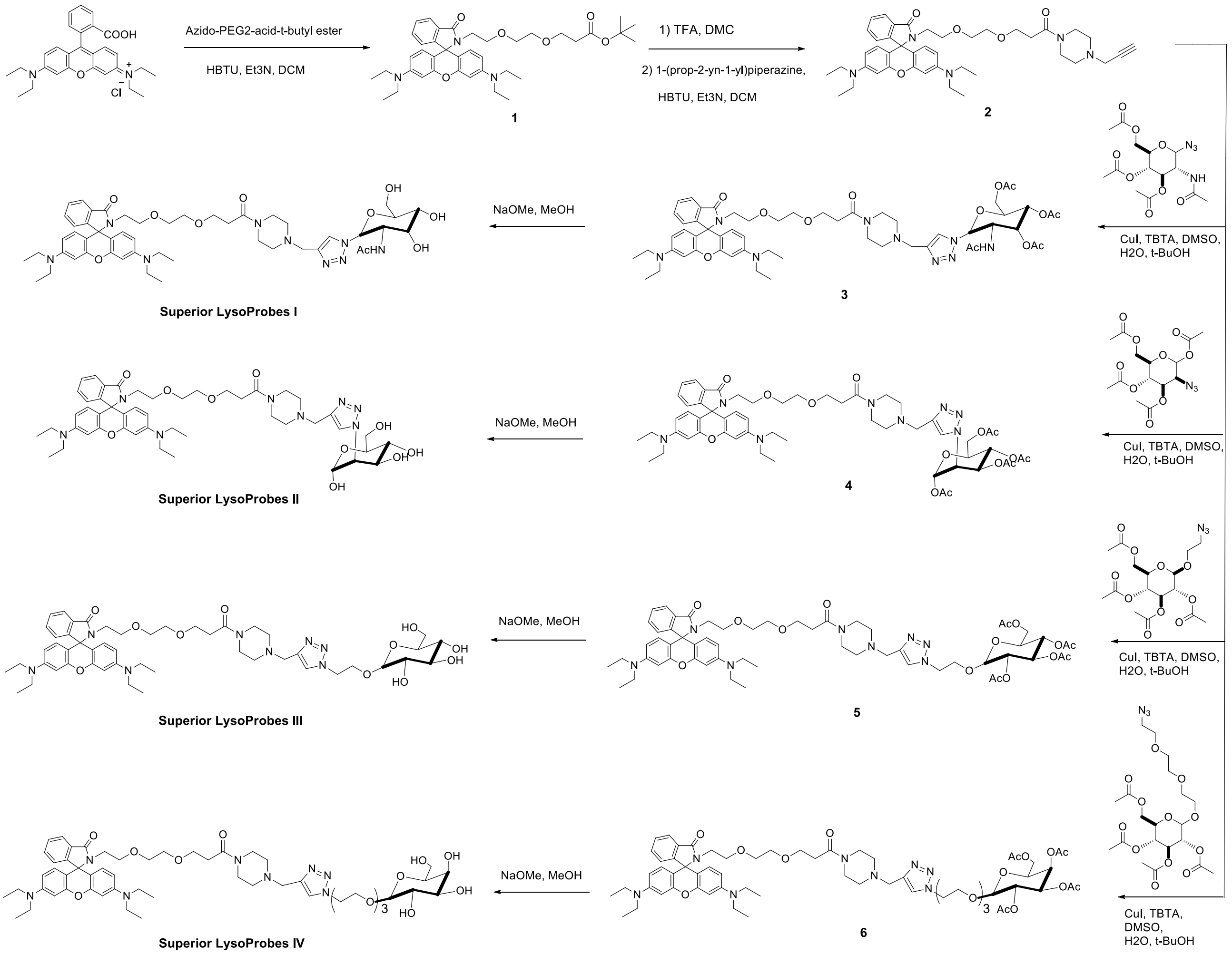

Scheme S1. Synthesis of Superior LysoProbes **I-IV**

Synthetic Procedure for compound 1

To a mixture of rhodamine B (481 mg, 1 mmol) and amine-PEG$_2$-acid tert-butyl ester (234 mg, 1 mmol) in DCM (20 mL), HBTU (758 mg, 2 mmol) and Et_3N (0.5 mL) were then added. The reaction mixture was stirred overnight at room temperature. The solvent was then removed by distillation under reduced pressure, and the crude residue was purified *via* flash chromatograph (EtOAc: Hexane, 2:1) to obtain the pure compound **1** as a colorless solid (420 mg, 64 %); ^{1}H NMR (400 MHz, $CDCl_3$) δ: 1.11 (m, 12H), 1.38 (s, 9H), 2.41 (m, 2H), 3.15 (m, 2H), 3.27-3.35 (m, 12H), 3.41 (m, 2H), 3.59 (m, 2H), 6.22 (d, 1H), 6.24 (d, 1H), 6.34 (d, 2H), 6.38 (s, 1H), 6.40 (s, 1H), 7.03 (m, 1H), 7.38 (m, 2H), 7.86 (m, 1H).

Synthetic Procedure for compound 2

To a stirred solution of compound **1** (410 mg, 0.62 mmol) in DCM (3 mL) at 0 ^oC was added TFA (3 mL). The reaction mixture was stirred overnight until TCL indicated that the starting material disappeared. After the solvent and TFA was removed by distillation under reduced pressure, the crude

residue was dissolved in DCM (10 ml) and sequentially added 1-(prop-2-yn-1-yl) piperazine trifluoroacetate (166 mg, 0.7 mmol), HBTU (416mg, 1.1 mmol) and Et_3N (0.5 mL). The reaction mixture was stirred for 6 h room temperature. The solvent was then removed by distillation under reduced pressure, and the crude residue was purified *via* flash chromatograph to obtain the colorless product **2** (403 mg, 91 %); ^1H NMR (400 MHz, $CDCl_3$) δ: 1.11 (m, 12H), 2.22 (m, 1H), 2.54 (m, 4H), 2.62 (m, 2H), 3.01(m, 2H), 3.27-3.36 (m, 14H), 3.47 (m, 4H), 3.67 (m, 4H), 6.20 (d, 1H), 6.23 (d, 1H), 6.34 (d, 2H), 6.37 (s, 1H), 6.39 (s, 1H), 7.03 (m, 1H), 7.40 (m, 2H), 7.87 (m, 1H).

Synthetic Procedure for compound 3

To a stirred solution of compound **2** (110 mg, 0.16 mmol) in DMSO: H_2O: *t*-BuOH (19 mL, 8:7:4) was sequentially added 2-Acetamido-3,4,6-tri-O-acetyl- 2-deoxy-β-D-glucopyranosyl azide (67 mg, 0.18 mmol), CuI and TBTA. The reaction was allowed to stir overnight at room temperature. The reaction mixture was washed with aqueous $NaHCO_3$ and then extracted with EtOAc. The combined organic layers were dried over Na_2SO_4 and filtered. The filtrate was concentrated to dryness under reduced pressure and the residue was purified *via* flash chromatograph (Acetone: MeOH, 30:1) to give the desired compound **3** as a colorless oil (90 mg，52 %). ^1H NMR (400 MHz, $CDCl_3$) δ: 1.06 (m, 12H), 1.62 (s, 3H), 1.94 (s, 3H), 1.96 (s, 3H), 1.98 (s, 3H), 2.35 (m, 4H), 2.46 (m, 2H), 3.06 (m, 2H), 3.21-3.26 (m, 12H), 3.35 (m, 4H), 3.51 (m, 2H), 3.59 (m, 4H), 3.93 (m, 1H), 4.03 (m, 1H), 4.21 (m, 1H), 4.51 (m, 1H), 5.11 (m, 1H), 5.39 (m, 1H), 5.98 (d, 1H), 6.18 (m, 2H), 6.27 (m, 2H), 6.31 (s, 1H), 6.33 (s, 1H), 6.96 (m, 1H), 7.33 (m, 3H), 7.74 (s, 1H), 7.78 (m, 1H).

Synthetic Procedure for compound 4

To a stirred solution of compound **2** (120 mg, 0.14 mmol) in DMSO: H_2O: *t*-BuOH (20 mL, 8:7:4) was sequentially added 2-acetamido-3,4,6-tri-O-acetyl-2-deoxy- β-D-glucopyranosyl azide (120 mg, 0.32 mmol), CuI and TBTA. The reaction was allowed to stir overnight at room temperature. The reaction mixture was washed with aqueous $NaHCO_3$ and then extracted with EtOAc. The combined organic layers were dried over Na_2SO_4 and filtered. The filtrate was concentrated to dryness under reduced pressure and the residue was purified *via* flash chromatograph (EtOAc: MeOH, 6:1) to give the desired compound **4** as a purple solid (47 mg, 24 %); ^1H NMR (400 MHz, $CDCl_3$) δ: 1.10 (m, 12H), 1.90 (s, 3H), 1.99 (s, 3H), 2.10 (s, 3H), 2.18 (s, 3H), 2.46 (m, 4H), 2.53 (t, J = 6.8 Hz, 2H), 3.08 (m, J = 7.2 Hz, 2H), 3.25-3.30 (m, 12H), 3.42 (m, 4H), 3.59 (m, 2H), 3.63-3.67 (m, 4H), 4.15 (m, 2H), 4.26 (m, 1H), 5.28 (t, J = 10.0 Hz, 1H), 5.33 (d, J = 4.8 Hz, 1H), 5.43 (m, 1H), 6.19 (m, 2H), 6.31-6.37 (m, 5H), 7.01 (m, 1H), 7.36 (m, 2H), 7.82 (m, 1H), 7.93 (s, 1H).

Synthetic Procedure for compound 5

To a stirred solution of compound **2** (144 mg, 0.2 mmol) in THF: H_2O: *t*-BuOH (20 mL, 3:1:1) was sequentially added 2-azidoethyl-2,3,4,6-tetra-O-acetyl-beta-D- glucopyranoside (85 mg, 0.2 mmol), Na L-ascorbate (100 mg, 0.5 mmol) and $CuSO_4$. The reaction was allowed to stir for 6h at room temperature. After evaporation, the residue was dissolved in EtOAc and washed with water. The combined organic layers were dried over Na_2SO_4 and filtered. The filtrate was concentrated to dryness under reduced pressure and the residue was purified *via* flash chromatograph (EtOAc: MeOH, 3:1) to give the desired compound **5** as a colorless solid (53 mg, 24%); ^{1}H NMR (400 MHz, $CDCl_3$) δ: 1.12 (m, 12H), 1.93 (s, 3H), 1.96 (s, 3H), 1.99 (s, 3H), 2.05 (s, 3H), 2.43 (m, 4H), 2.53 (t, J = 7.2 Hz, 2H), 3.13 (m, J = 7.2 Hz, 2H), 3.27-3.33 (m, 12H), 3.40 (m, 4H), 3.56 (m, 2H), 3.63-3.69 (m, 5H), 3.90 (m, 1H), 4.08-4.11 (m, 1H), 4.18-4.23 (m, 2H), 4.44-4.60 (m, 3H), 4.94 (m, 1H), 5.03 (t, J = 9.6 Hz, 1H), 5.14 (t, J = 9.6 Hz, 1H), 6.21 (m, 2H), 6.33 (m, 2H), 6.37 (s, 1H), 6.39 (s, 1H), 7.03 (m, 1H), 7.37 (m, 2H), 7.50 (s, 1H), 7.84 (m, 1H).

Synthetic Procedure for compound 6

To a stirred solution of compound **2** (130 mg, 0.18 mmol) in THF: H_2O: *t*-BuOH (20 mL, 3:1:1) was sequentially added 2-[2-(2-Azidoethoxy)ethoxy]ethyl 2,3,4,6-Tetra-O-acetyl-D-galactopyranoside (93 mg, 0.18 mmol), Na L-ascorbate (100 mg, 0.5 mmol) and $CuSO_4$. The reaction was allowed to stir for 6h at room temperature. After evaporation, the residue was dissolved in EtOAc and washed with water. The combined organic layers were dried over Na_2SO_4 and filtered. The filtrate was concentrated to dryness under reduced pressure and the residue was purified *via* flash chromatograph (EtOAc: MeOH, 2:1) to give the desired compound **6** as a colorless solid (59 mg, 27 %); δ: 1.13 (m, 12H), 1.95 (s, 3H), 2.00 (m, 6H), 2.11 (s, 3H), 2.41 (m, 4H), 2.53 (t, J = 7.2 Hz, 2H), 3.14 (m, J = 7.2 Hz, 2H), 3.27-3.33 (m, 12H), 3.41 (m, 4H), 3.56-3.60 (m, 8H), 3.64-3.68 (m, 5H), 3.85 (m, J = 5.6 Hz, 2H), 3.86-3.96 (m, 2H), 4.06-4.17 (m, 2H), 4.52 (m, 3H), 5.01 (m, 1H), 5.16 (m, 1H), 5.35 (m, 1H), 6.21 (m, 2H), 6.33 (m, 2H), 6.37 (s, 1H), 6.39 (s, 1H), 7.03 (m, 1H), 7.38 (m, 2H), 7.60 (s, 1H), 7.84 (m, 1H).

General procedure for the synthesis of Superior LysoProbe I-IV

The appropriate protected compound **3-6** was dissolved in MeOH (5 mL). Freshly prepared NaOMe solution (1 M in MeOH) was added until pH 9 was reached. Then the reaction mixture was stirred at room temperature until the deprotection reaction was complete. After evaporation of the solvent under reduced pressure, the residue was purified by column chromatography to give the Superior LysoProbe **I-IV**.

Superior LysoProbe I: ^{1}H NMR (400 MHz, CD_3OD) δ: 1.13 (t, J = 6.8 Hz, 12H), 1.74 (s, 3H), 2.41-2.48 (m, 4H), 2.55 (t, J = 6.4 Hz, 2H), 3.06 (t, J = 6.4 Hz, 2H), 3.23 (m, 2H), 3.33-3.38 (m, 12H), 3.49-3.68 (m, 16H), 3.75 (m, 1H), 3.87 (m, 1H), 4.21 (t, J = 10.0 Hz, 1H), 5.73 (d, J = 10.0 Hz, 1H), 6.34 (s,

4H), 6.41 (s, 2H), 7.03 (m, 1H), 7.50 (m, 2H), 7.83 (m, 1H), 8.06 (m, 1H). ^{13}C NMR (100 MHz, CD_3OD) δ: 172.0,170.7, 168.9, 153.9, 153.5, 149.2, 132.9, 130.7, 128.5, 128.3, 127.6, 123.9, 122.9, 122.3, 108.3, 104.9, 97.8, 87.1, 86.0, 80.0, 74.5, 70.1, 69.8, 67.9, 67.1, 65.7, 61.1, 55.5, 44.1, 39.2, 33.0, 21.5, 11.7.

Superior LysoProbe III: ^{1}H NMR (400 MHz, CD_3OD) δ: 1.12 (t, J = 6.8 Hz, 12H), 2.42-2.46 (m, 4H), 2.55 (t, J = 6.0 Hz, 2H), 3.05 (t, J = 6.4 Hz, 2H), 3.15 (m, 1H), 3.23-3.36 (m, 20H), 3.49 (m, 4H), 3.63 (m, 6H), 3.83 (d, J = 12 Hz, 1H), 3.99 (m, 1H), 4.21 (m, 1H), 4.26 (d, J = 8 Hz, 1H), 4.62 (m, 2H), 6.32 (s, 4H), 6.40 (s, 2H), 7.01 (m, 1H), 7.49 (m, 2H), 7.84 (m, 1H), 8.02 (m, 1H). ^{13}C NMR (100 MHz, CD_3OD) δ: 170.7, 169.0, 153.9, 153.5, 149.1, 144.1, 132.9, 130.7, 128.5, 125.3, 123.8, 122.3, 108.3, 104.9, 103.3, 97.8, 76.8, 76.7, 73.7, 70.3, 69.7, 67.8, 67.7, 67.0, 65.7, 61.5, 52.8, 52.1, 50.4, 45.5, 44.1, 41.3, 39.3, 33.0, 11.7.

Superior LysoProbe IV: ^{1}H NMR (400 MHz, CD_3OD) δ: 1.13 (t, J = 6.8 Hz, 12H), 2.42-2.46 (m, 4H), 2.55 (t, J = 6.4 Hz, 2H), 3.05 (t, J = 6.4 Hz, 2H), 3.23 (m, 2H), 3.32-3.37 (m, 12H), 3.44 (m, 4H), 3.57-3.66 (m, 16H), 3.71 (m, 4H), 3.82 (m, 1H), 3.87 (m, 2H), 3.98 (m, 1H), 4.22 (d, J = 7.6 Hz, 1H), 4.55 (t, J = 4.8 Hz, 2H), 6.33 (s, 4H), 6.41 (s, 2H), 7.03 (m, 1H), 7.49 (m, 2H), 7.83 (m, 1H), 7.95 (m, 1H).

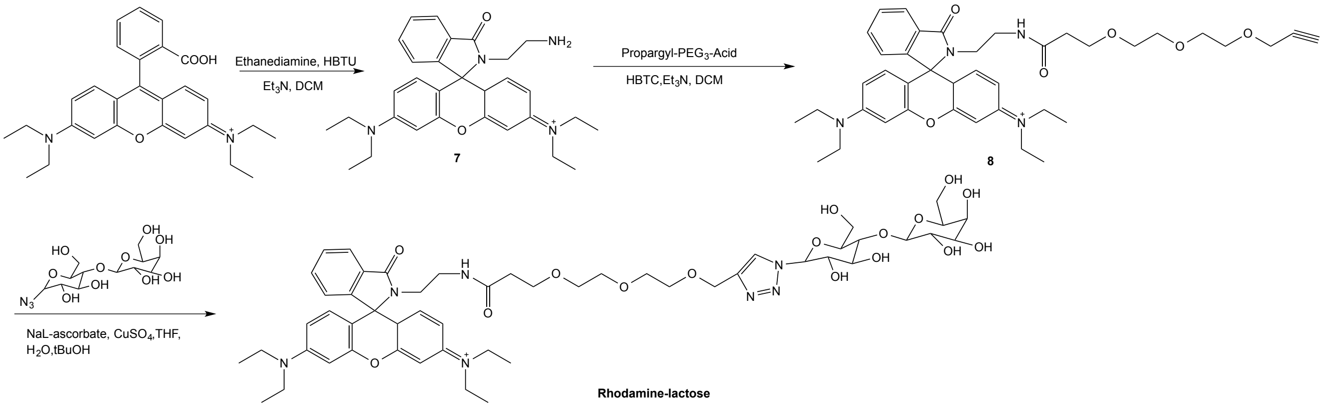

Scheme S2. Synthesis of rhodamine-lactose

Synthetic Procedure for compound 7:

To A mixture of Rhodamine B (960 mg, 2 mmol) and Ethanediamine (5 mL) in DCM (40 mL) was sequentially added HBTU (1200 mg, 3.2 mmol) and Et_3N (3 mL). The reaction mixture was stirred for 48h at room temperature. The solvent was then removed by distillation under reduced pressure, and the crude residue was purified *via* flash chromatograph (EtOAc: Hexane: MeOH: Et_3N, 20:10:2:1) to obtain the pure product **7** as a colorless solid (697 mg, 72 %); ^{1}H NMR (400 MHz, $CDCl_3$) δ: 1.08 (t, 12H), 2.35

(m, 2H), 3.12 (t, 2H), 3.27(m, 8H), 6.19 (d, 1H), 6.22 (d, 1H), 6.31 (d, 2H), 6.36 (s, 1H), 6.38 (s, 1H), 7.00 (m, 1H), 7.35 (m, 2H), 7.85 (m, 1H).

Synthetic Procedure for compound 8:

To A mixture of **7** (532 mg, 1.1 mmol) and Propargyl-PEG3-Acid (216 mg, 1.0 mmol) in DCM (15 mL) was sequentially added HBTU (450 mg, 1.2 mmol) and Et_3N (1 mL). The reaction mixture was stirred overnight at room temperature. The solvent was then removed by distillation under reduced pressure, and the crude residue was purified *via* flash chromatograph (EtOAc: Hexane: MeOH, 16:4:1) to obtain the pure product **8** as a purple solid (540 mg, 77 %); ^{1}H NMR (400 MHz, $CDCl_3$) δ: 1.12 (t, 12H), 2.40 (m, 3H), 2.88 (m, 2H), 3.31(m, 10H), 3.62-3.75 (m, 10H), 4.25(m, 2H), 6.27 -6.29 (m, 2H), 6.35 (d, 2H), 6.39 (s, 1H), 6.42 (s, 1H), 6.70 (m, 1H, NH), 7.06 (m, 1H), 7.43 (m, 2H), 7.86 (m, 1H).

Synthetic Procedure for rhodamine-lactose:

The compound **8** (135 mg, 0.20 mmol), sodium ascorbate (36 mg, 0.2 mmol) and copper sulfate (6 mg, 0.02mmol) were added to 1-azido-β-(D)-lactopyranoside (90 mg, 0.25 mmol) in 5 mL of THF/ H_2O/*t*-BuOH mixture (3/1/1). After stirring overnight at room temperature, the solvent was evaporated and the residue was purified *via* flash chromatograph (DCM/methanol, increasing from 90/10 to 80/20) to give the desired rhodamine-lactose as a colorless solid (87 mg, 41 %).^{1}H NMR (400 MHz, $CDCl_3$) δ: 1.06 (t, J = 6.4 Hz, 12H), 2.22 (m, 2H), 2.84 (m, 2H), 3.18 (m, 2H), 3.25 (m, 10H), 3.33 (m, 10H), 3.42 (m, 8H), 3.53 (m, 4H), 3.64-3.86 (m, 9H), 4.44 (m, 2H), 4.83 (m, 1H), 5.64 (m, 1H), 6.22 (m, 2H), 6.38 (m, 4H), 6.82 (m, 1H), 6.99 (m, 1H), 7.43 (m, 2H), 7.83 (m, 1H), 8.01 (s, 1H).

Spectral Properties of Probes I-IV:

Fluorescence spectra were obtained with a Horiba Jobin Yvon Fluoromax-4 spectrofluorometer using an excitation wavelength of 480nm and slit widths of 5 nm. Fluorescence cells with optical pathlength of 10x10 mm were used.

1 μM of Superior LysoProbes I-IV were prepared by diluting a DMSO solution of probes I-IV (1mM) 1000 times with citric acid (0.1M)-potassium phosphate dibasic (0.2M) buffers. The pH response of Superior LysoProbe **I-IV** (1 μM) was tested in potassium phosphate-citric acid buffer with various pH values, respectively.

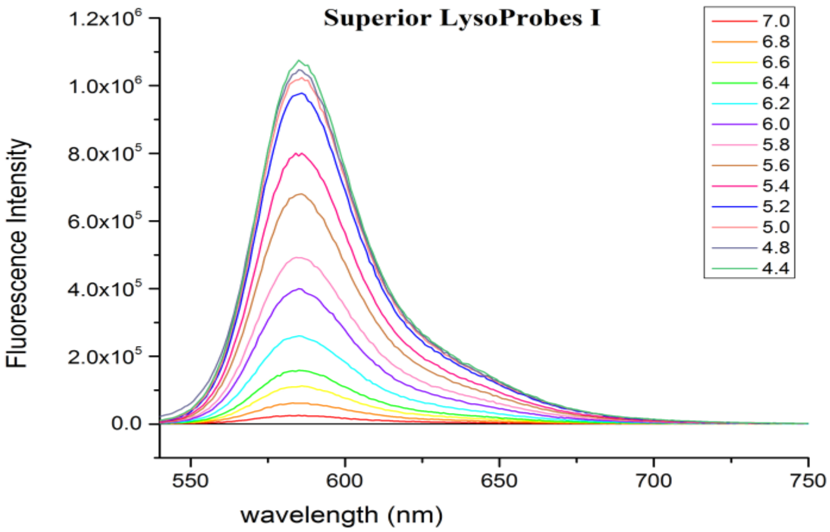

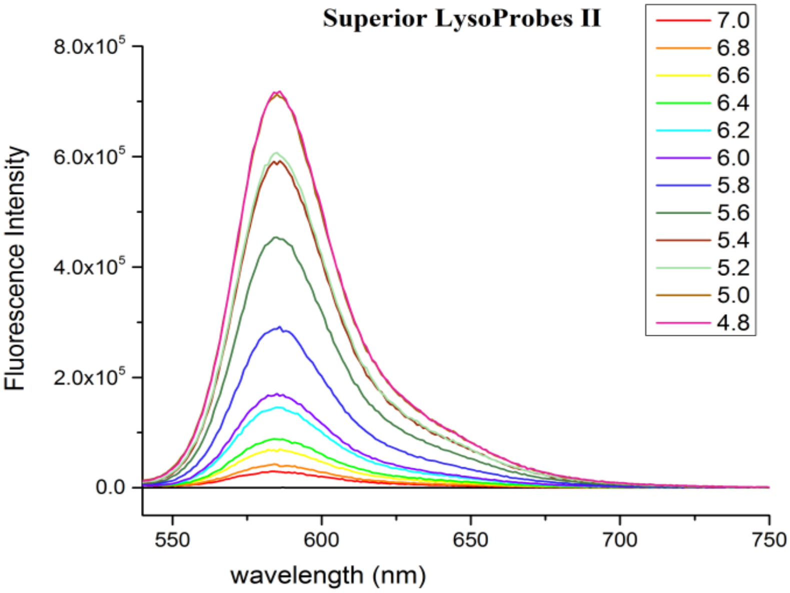
Superior LysoProbes II
7.0
6.8
6.6
6.4
6.2
6.0
5.8
5.6
5.4
5.2
5.0
4.8
8.0x10^5
6.0x10^5
4.0x10^5
2.0x10^5
0.0
Fluorescence Intensity
550
600
650
700
750
wavelength (nm)

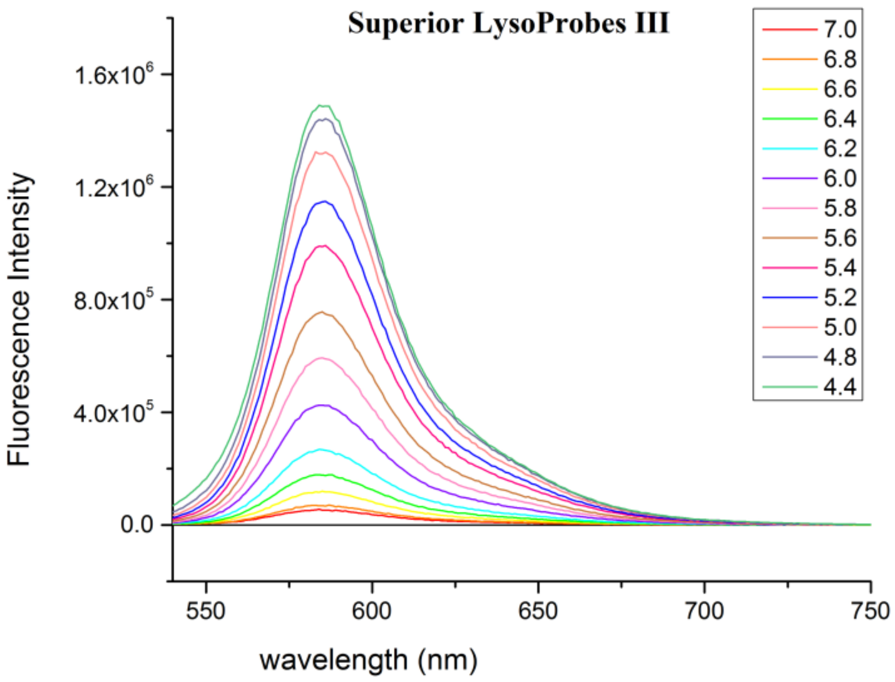

Superior LysoProbes III
7.0
6.8
6.6
6.4
6.2
6.0
5.8
5.6
5.4
5.2
5.0
4.8
4.4
Fluorescence Intensity
1.6x10^6
1.2x10^6
8.0x10^5
4.0x10^5
0.0
550
600
650
700
750
wavelength (nm)

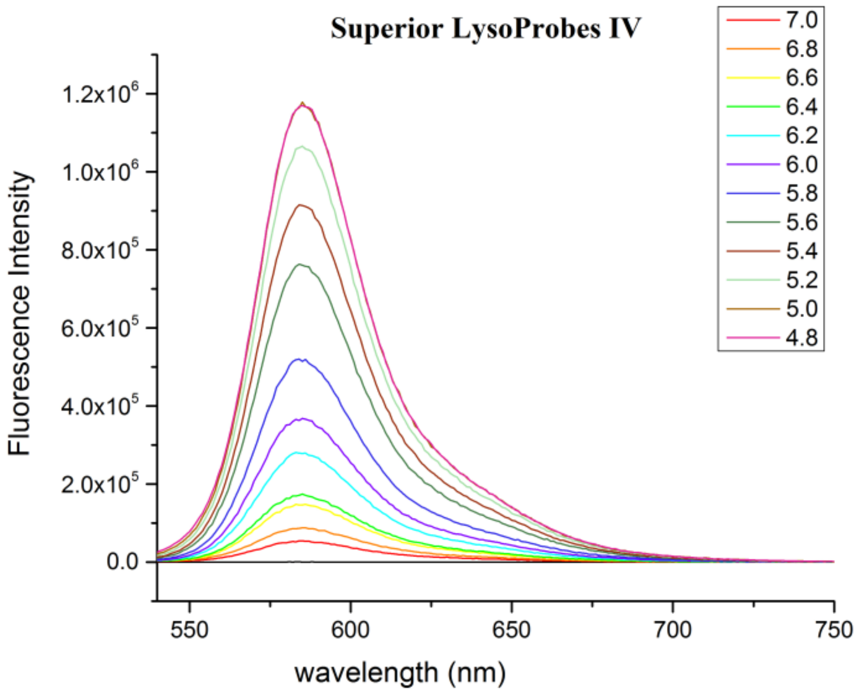

Figure S1. The pH response of Superior LysoProbe **I-IV** (1 μM) was tested in potassium phosphate-citric acid buffer with various pH values, respectively.

Cell Culture:

HeLa cells (CCL-2) were obtained from American Type Cell Culture collection (ATCC) and grown in Eagle's Minimal Essential Medium (EMEM) and 10% FBS (Sigma-Aldrich, heat inactivated) and incubated at 37°C in 5% CO_2. One or two days before imaging, the cells were passaged and plated in phenol red-free medium on 4-well chamber slides (Corning, Corning NY), and allowed to grow to 50–70% confluence. The retinal pigment epithelial cell line ARPE-19 was obtained from ATCC and grown in DMEM/Ham's F12 1:1 (Hyclone, Fisher Sci.) containing 10% FBS (Sigma-Aldrich, heat inactivated). ARPE-19 (50.000 cells/well) cells were grown in 24-well plates for 12 h. RBE cells were obtained from ATCC and cells were maintained in RPMI-1640 (Gibco BRL, Carlsbad, CA, USA) supplemented with 10% fetal bovine serum (Gibco) and 100 unites/mL penicillin and 0.1 mg/mL streptomycin (Penicillin and Streptomycin Solution (100X), Gibco, USA) at 37 °C in a humidified atmosphere containing 5% CO_2. CW-2 cells were obtained from ATCC and cultured in Dulbecco's modified Eagle's medium supplemented with 10% heat-inactivated fetal bovine serum, penicillin (final concentration, 100 U/ml), and streptomycin (final concentration, 0.1 mg/ml), in a humidified atmosphere of 5% CO_2 and 95% air at 37 °C. HGC-27cells were obtained from ATCC and maintained in RPMI 1640 medium containing 10% FBS, 100 U/mL penicillin, and 100 U/mL streptomycin at 37C with 5% CO2. Cells were passaged at 80% confluency using 1mmol/L ethylene diamine tetraacetic acid (EDTA)-0.025% trypsin. MCF-7 cell line obtained from ATCC and maintained as an attached monolayer culture in the commercially defined RPMI 1640 medium, supplemented with 10% (v/v) heat-inactivated fetal bovine serum (FBS), 2 mM l-glutamine, 100 U/mL and 100 μg/mL

penicillin–streptomycin, and 25 μM 4-(2-hydroxyethyl)-1-piperazineethanesulfonic acid (HEPES).

Co-localization study of living cells: Cells were adjusted to the density of 50000 per cm^2, incubated for 48h, and then washed with cell culture medium twice. For the Superior LysoProbe and LysoTracker co-stain, solution of Superior LysoProbe I-IV (1 μM) and LysoTracker Green (2 μM) in cell culture medium were added to pre-washed cells and incubated at 37°C for 45 min, respectively. For the Superior LysoProbe I-IV and MitoTracker co-stain, solutions of Superior LysoProbe I-IV (1 μM) and MitoTracker (80 nM) in cell culture medium were added to the pre-washed cells and incubated at 37°C for 30 min, respectively. For nuclear staining, cells were incubated with 1 μM Hoechst at 37°C for 15 min prior to imaging. Confocal fluorescence imaging studies were performed with Olympus laser-scanning microscope with a 60× oil-immersion objective lens. Image analysis was performed using ImageJ (National Institute of Health). For quantitative analysis of fluorescence of the confocal images, threshold of the images was set to 10, area of fluorescence was selected with the "create selection" function and fluorescent intensity of the whole image was measured.

For intracellular pH measurement assay: ARPE-19 cells were treated with 10 μM nigericin and equilibrated for 2 min with intracellular pH calibration buffer kit (Life Technologies) prior to image acquirement by following our previous published procedure.[28]

Cell Imaging Data:

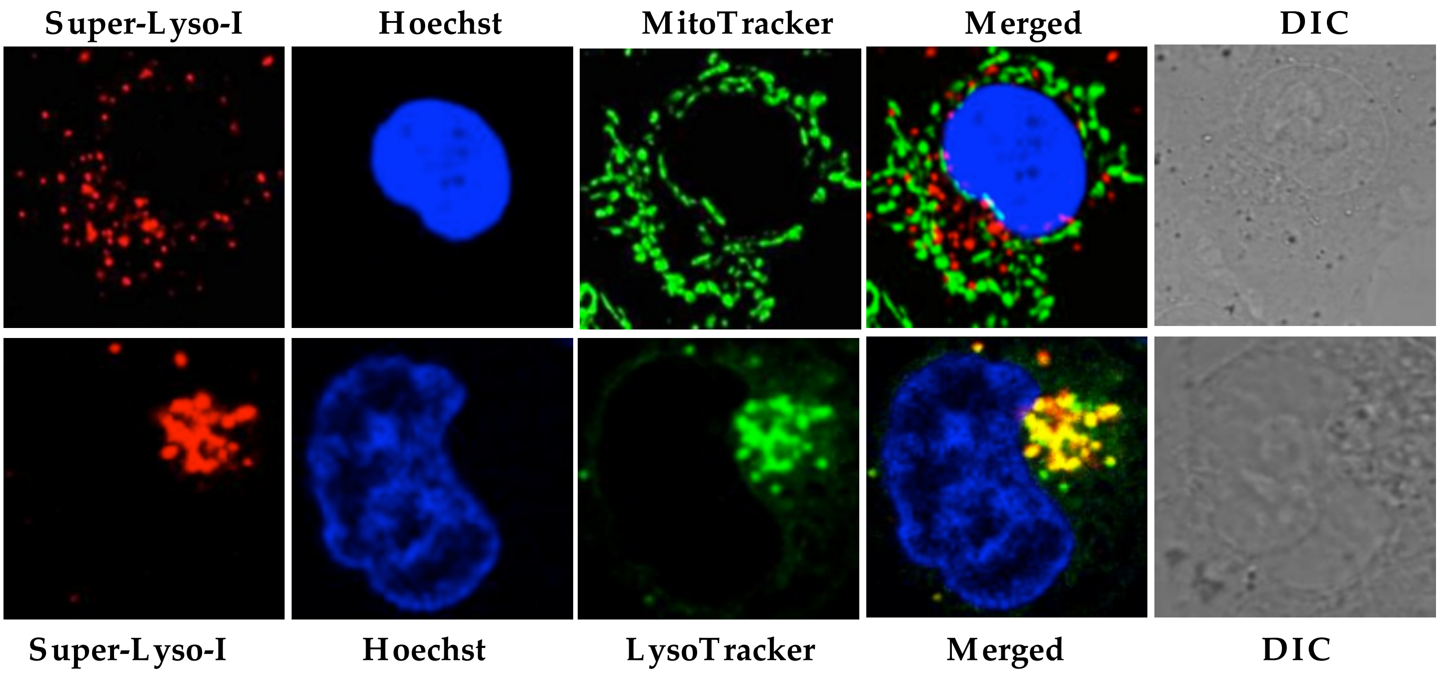

Figure S2. Intracellular distribution of Superior LysoProbe I (1 μM) compared to MitoTracker (80 nM) and LysoTracker Green (2 μM). HeLa cells were imaged on an inverted laser scanning fluorescent microscope (Olympus) using a 60 x oil immersion objective lens.

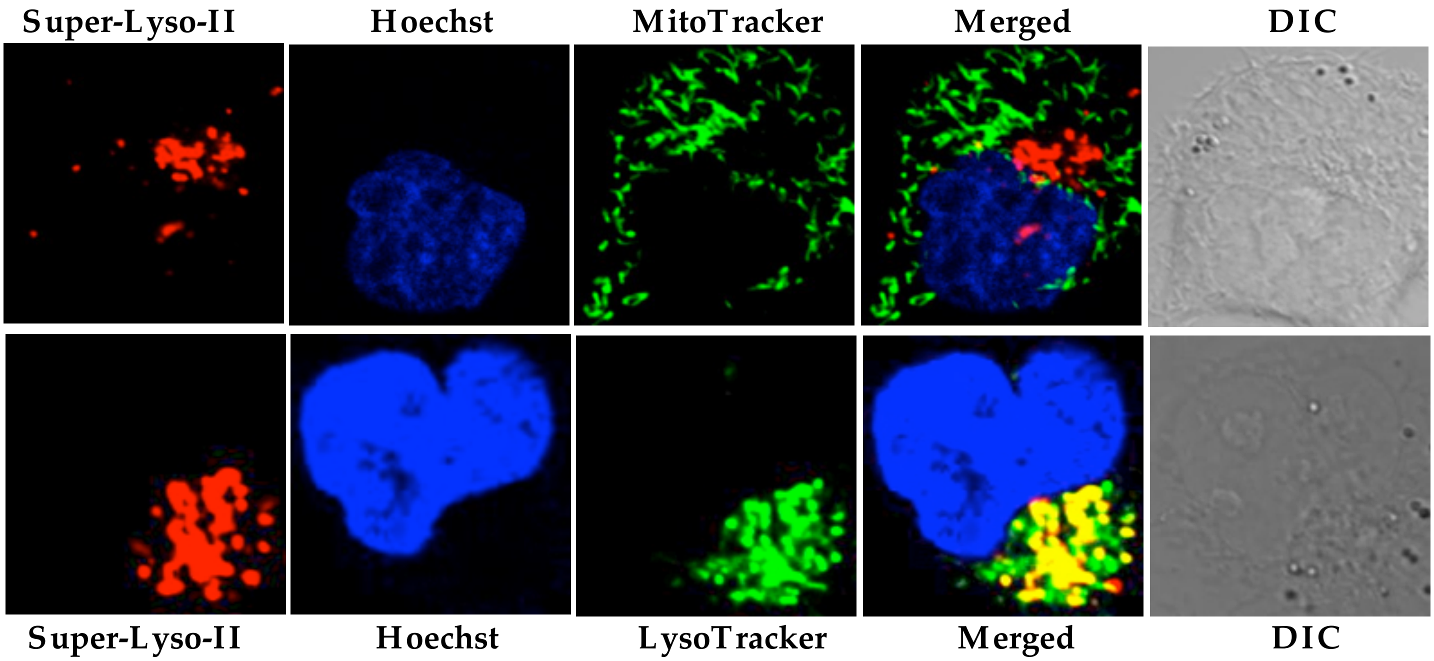

Figure S3. Intracellular distribution of Superior LysoProbe II (1 μM) compared to MitoTracker (80 nM) and LysoTracker Green (2 μM). HeLa cells were imaged on an inverted laser scanning fluorescent microscope (Olympus) using a 60 x oil immersion objective lens.

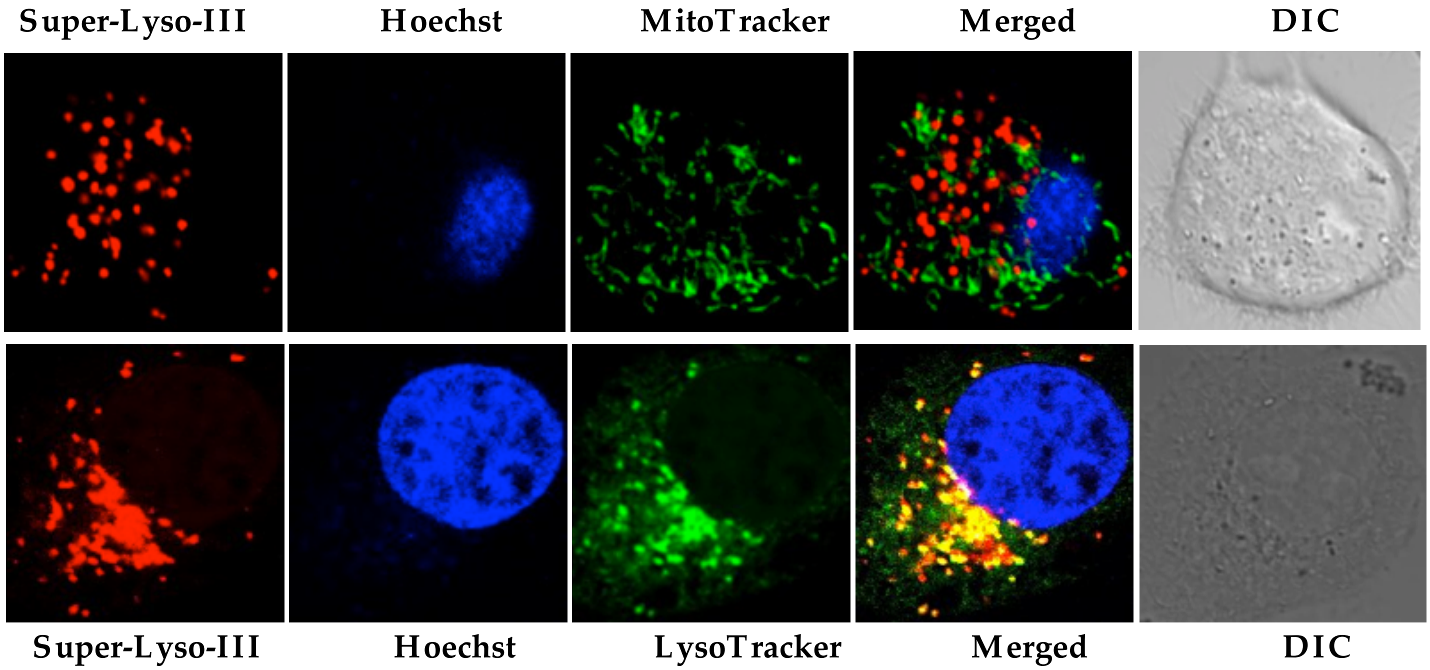

Figure S4. Intracellular distribution of Superior LysoProbe III (1 μM) compared to MitoTracker (80 nM) and LysoTracker Green (2 μM). HeLa cells were imaged on an inverted laser scanning fluorescent microscope (Olympus) using a 60 x oil immersion objective lens.

Table S1: Quantifying Colocalization Measurement by Correlation

Regions of interest (ROI)	PCC	MOC
Superior-LysoProbe **I**	0.65 ± 0.04	0.72 ± 0.02
Superior-LysoProbe **II**	0.69 ± 0.03	0.71 ± 0.05
Superior-LysoProbe **III**	0.67 ± 0.05	0.74 ± 0.04
Superior-LysoProbe **IV**	0.71 ± 0.04	0.75 ± 0.03

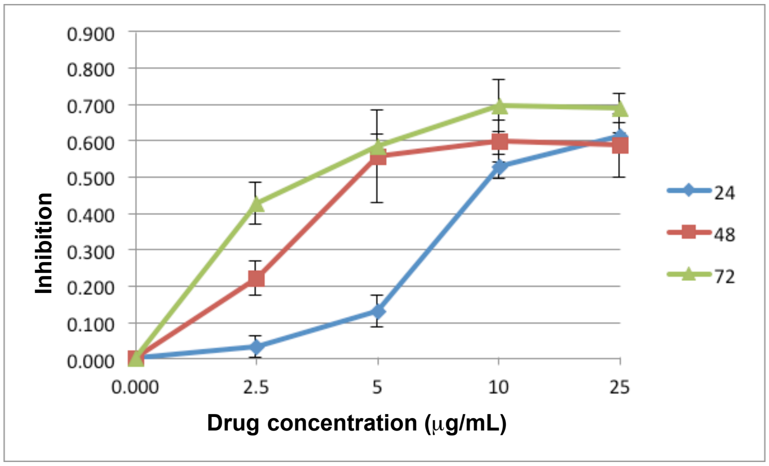

Figure S5. Influence of lobaplatin treatment on the cell proliferation in human cholangiocarcinoma RBE cells.

(A)

(B)

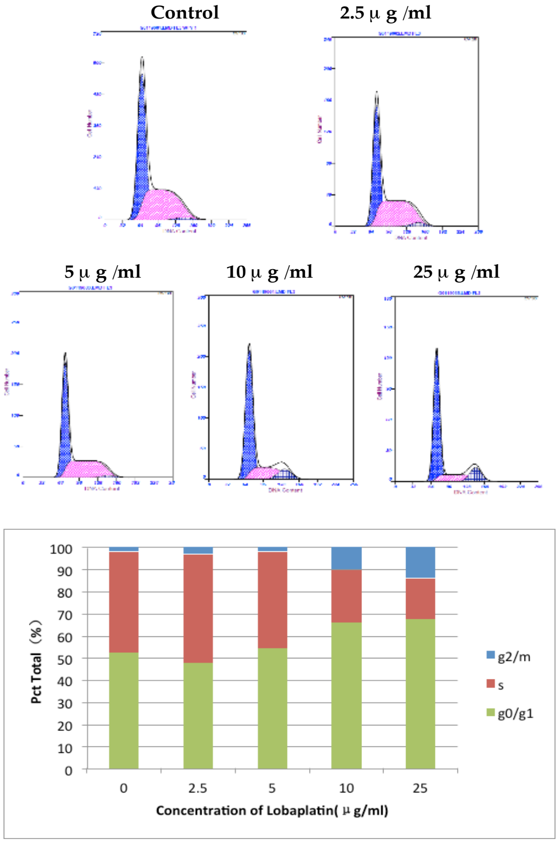

Figure S6. Influence of lobaplatin treatment upon cell cycle progression in human cholangiocarcinoma RBE cells. (A) RBE cells were exposed to various concentrations of lobaplatin (2.5, 5, 10 and 25μg/mL) for 24 hours followed by analysis of cell cycle by flow cytometry; (B) distribution of RBE cells at different phases of the cell cycle distribution. All assays were done in triplicate.

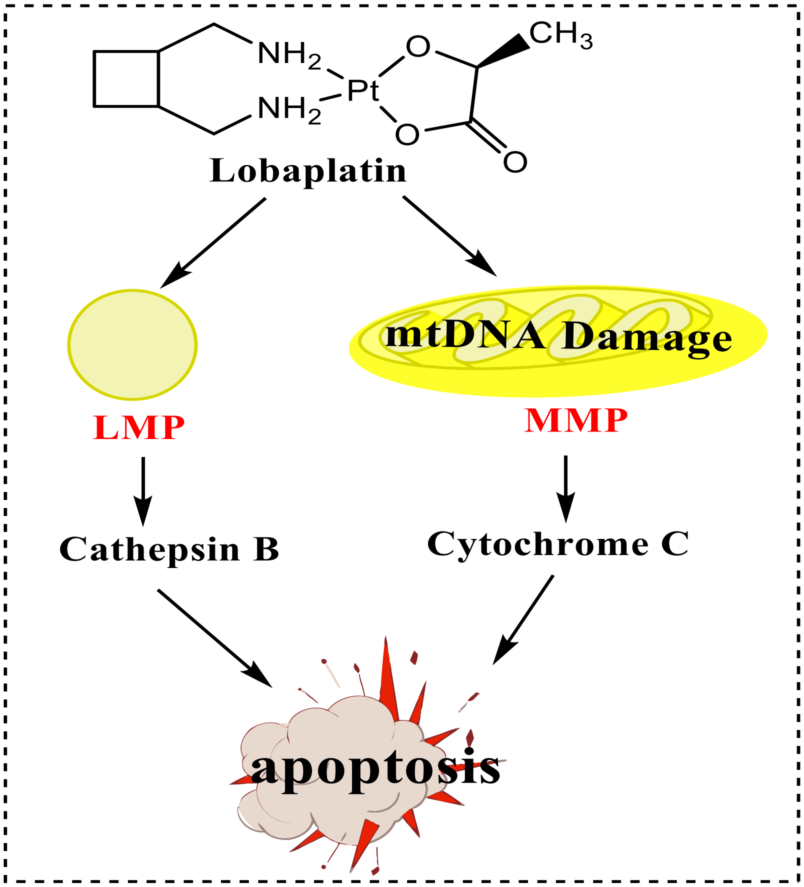

Figure S7. Proposed machenism of lobaplatin in RBE cells. Lobaplatin-induced cell apoptosis may involve both mitochondrial damage and lysosomal membrane destabilization. The coordination between mitochondria and lysosomes in apoptosis needs to be further investigated.

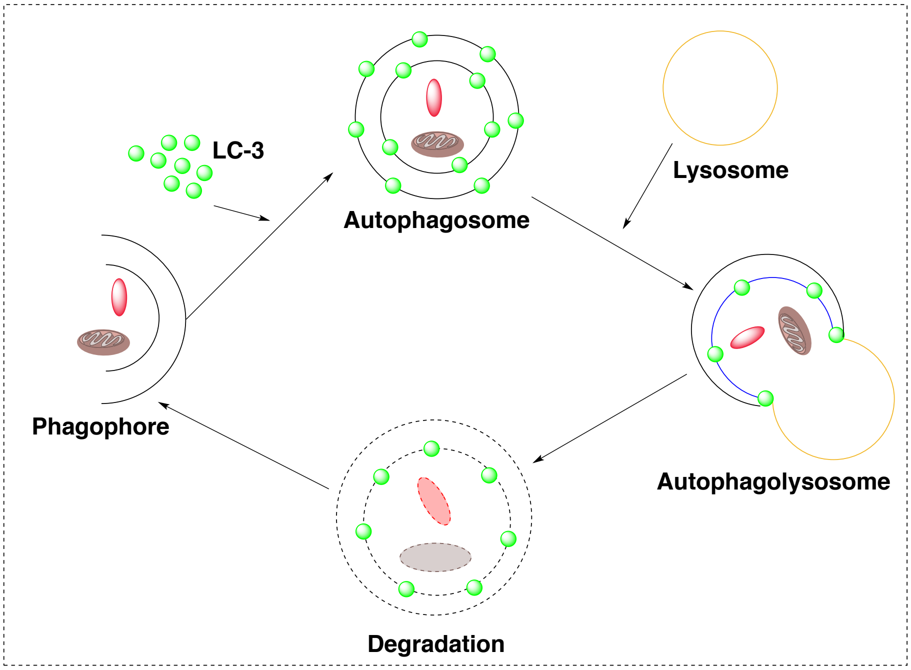

Figure S8. Schematic model of the steps involved in autophagy. Schematic drawing showing the autophagy process with phagophore and autophagosome formation followed by fusion of autophagosomes to lysosomes to form autophagolysosomes.

NMR Spectra:

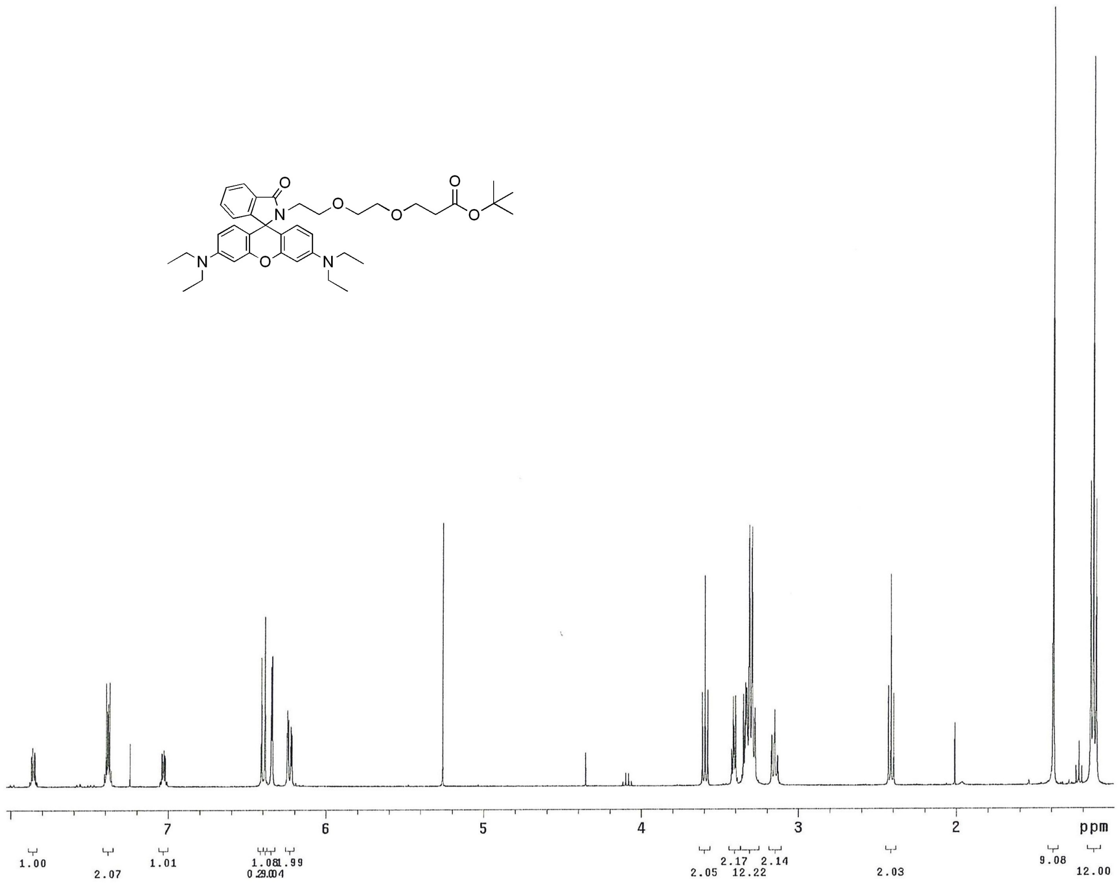

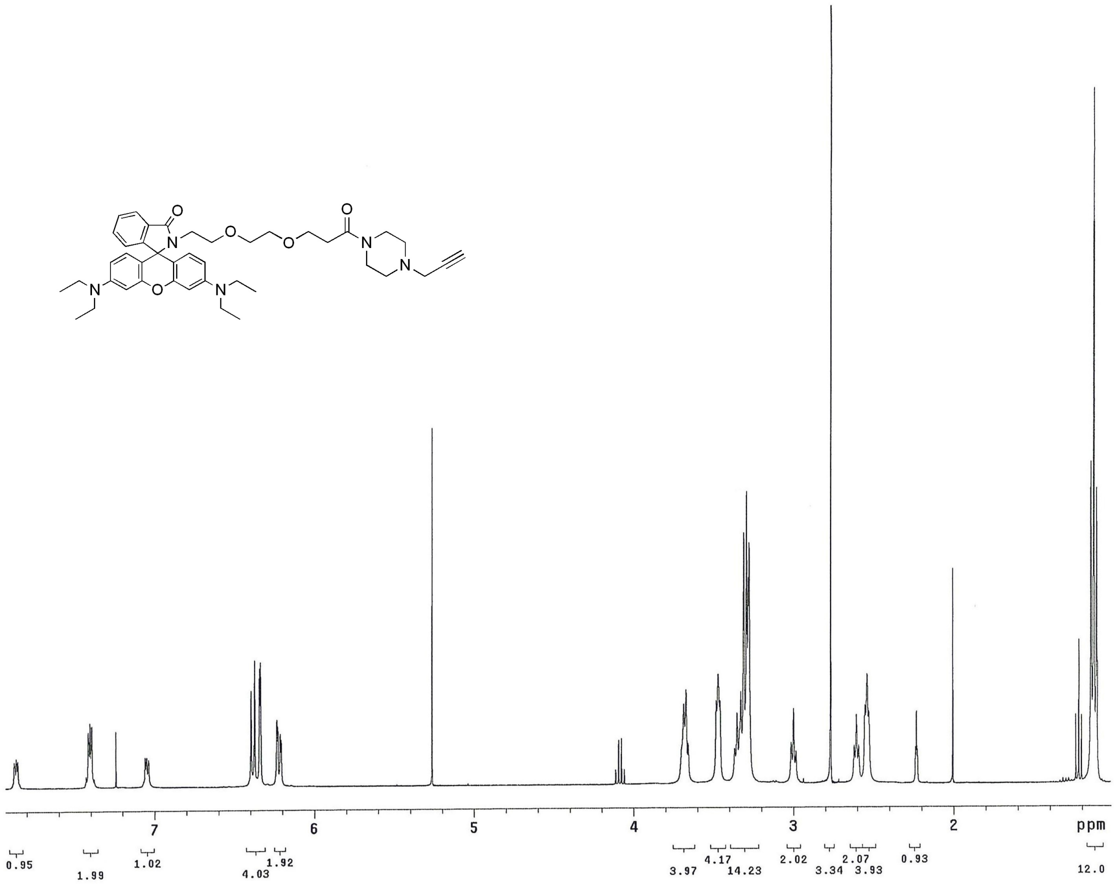
O
N
O
O
O
N
N
N
O
N
7
6
5
4
3
2
ppm
0.95
1.99
1.02
4.03
1.92
3.97
4.17
14.23
2.02
3.34
2.07
3.93
0.93
12.0

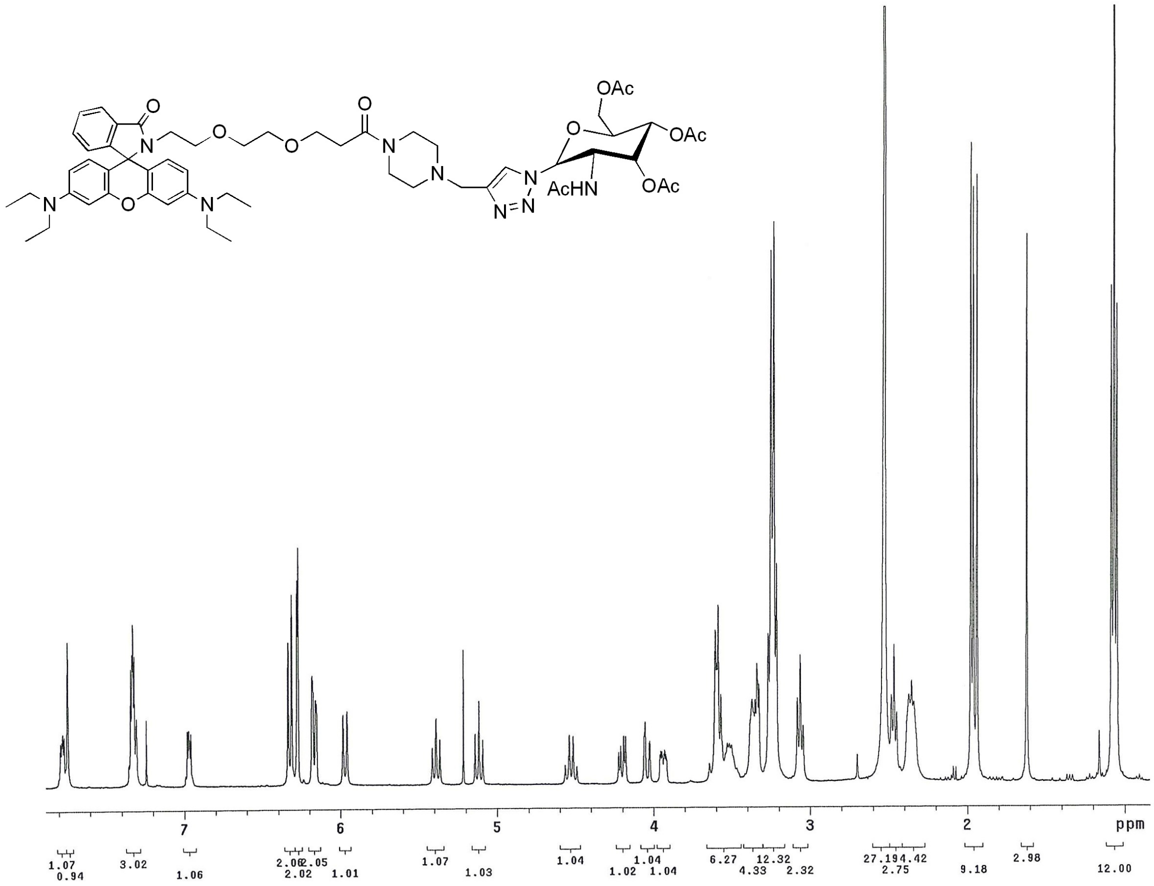
OAc
OAc
OAc
AcHN
7
6
5
4
3
2
ppm
1.07
0.94
3.02
1.06
2.06
2.02
2.05
1.01
1.07
1.03
1.04
1.02
1.04
1.04
6.27
4.33
12.32
2.32
27.19
2.75
4.42
9.18
2.98
12.00

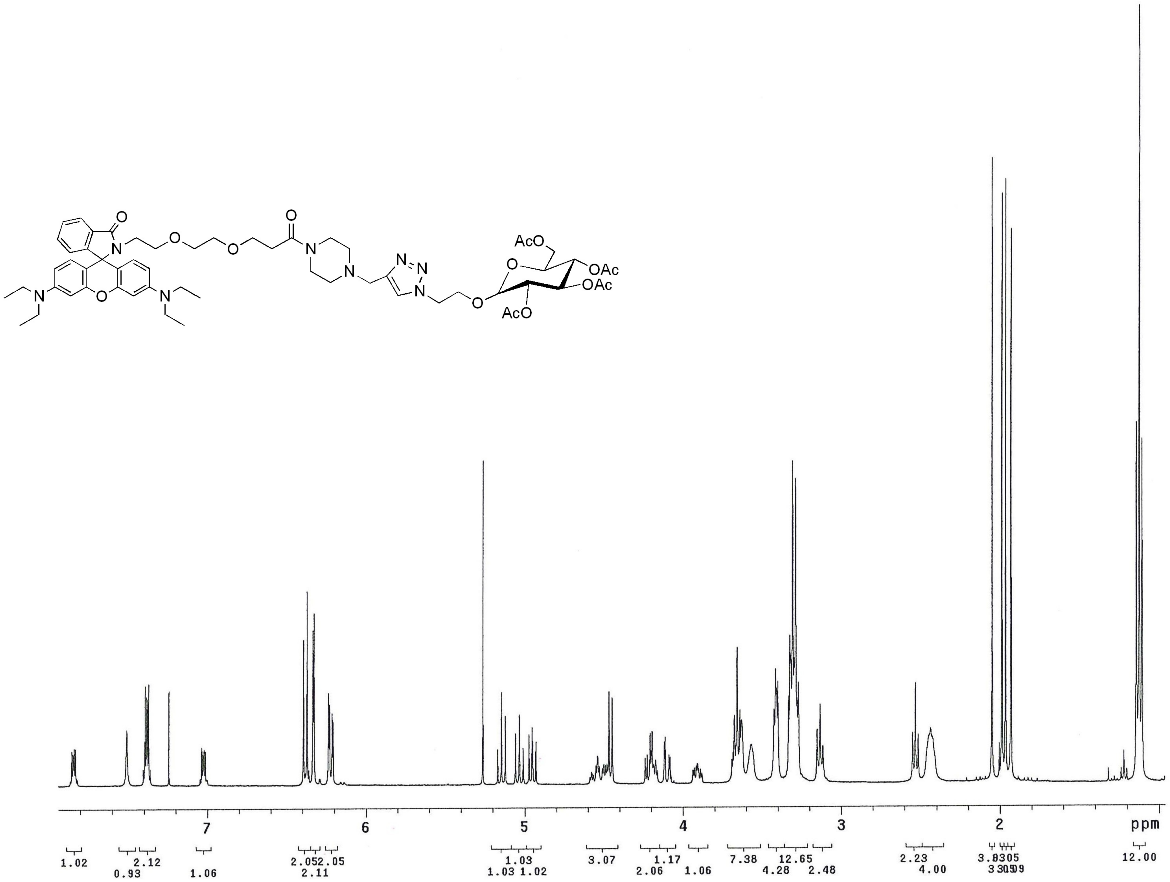

ppm

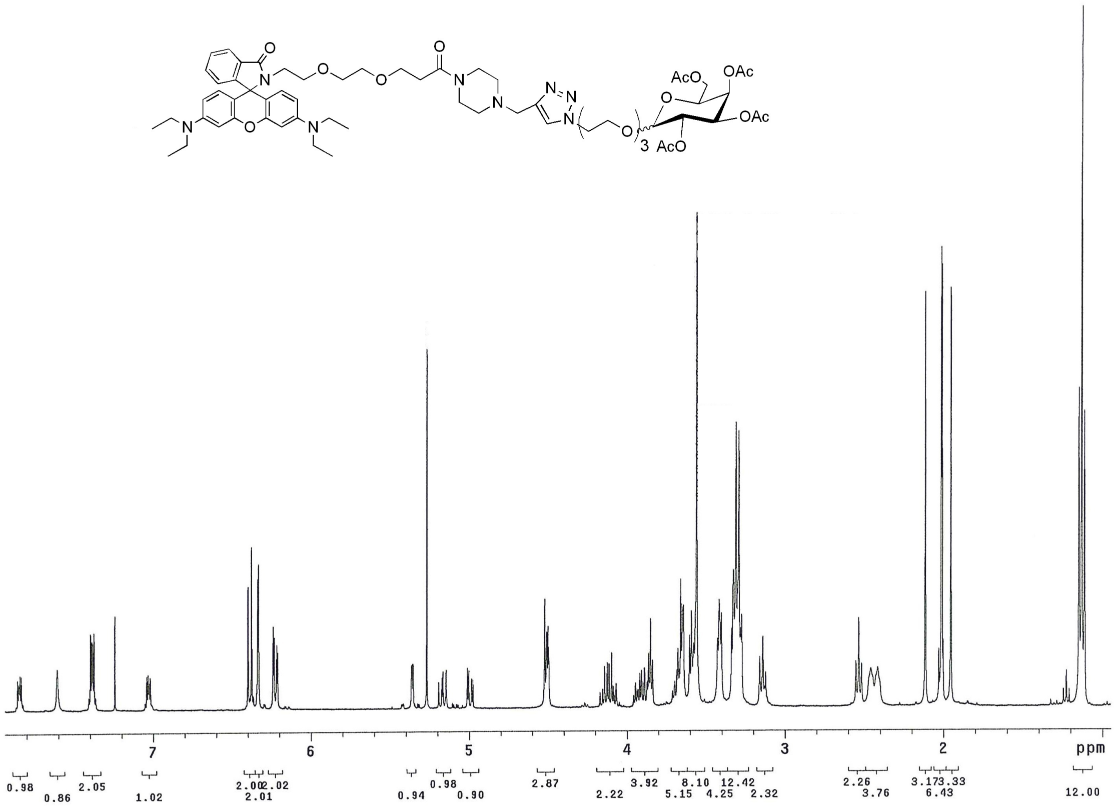

O
N
O
O
O
N
N
N
N
N
N
N
O
O
3
AcO
OAc
OAc
AcO
N
O
N
7
6
5
4
3
2
ppm
0.98
0.86
2.05
1.02
2.00
2.01
2.02
0.94
0.98
0.90
2.87
2.22
3.92
5.15
8.10
4.25
12.42
2.32
2.26
3.76
3.17
6.43
3.33
12.00

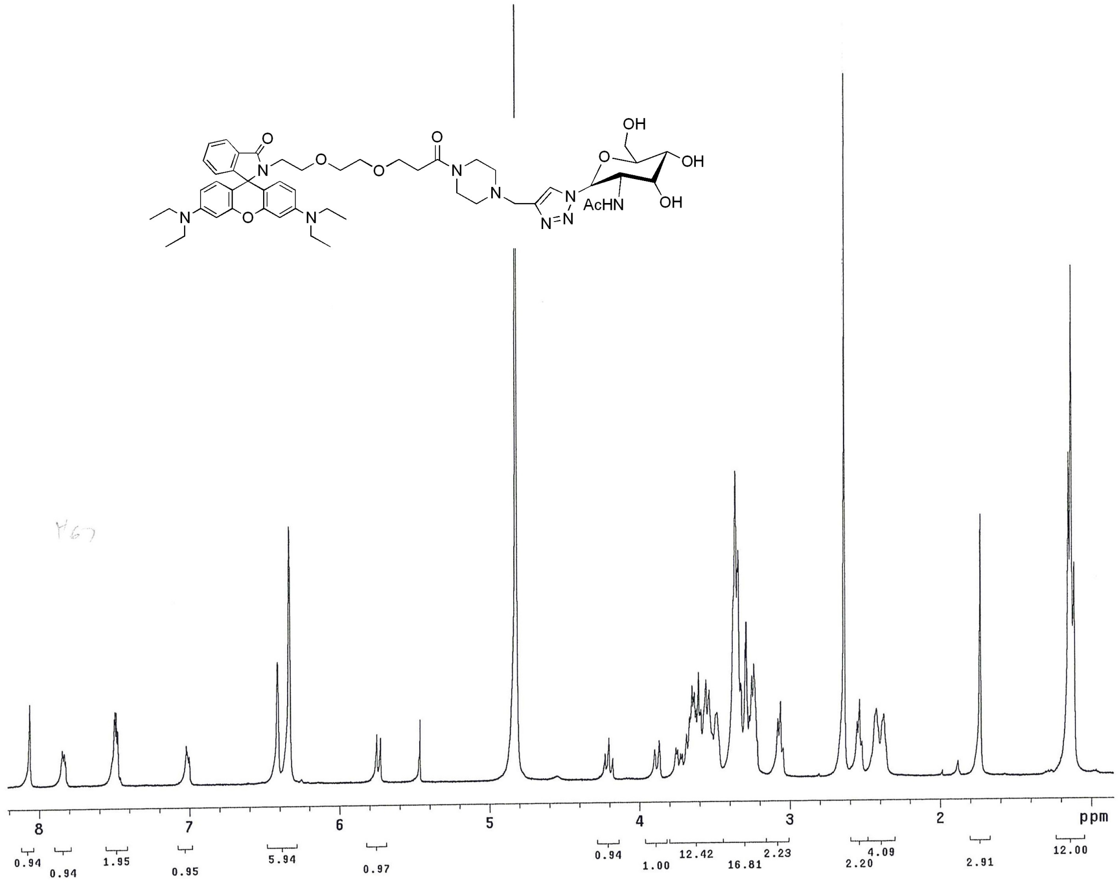
OH
OH
OH
O
AcHN
N
N=N
O
O
O
N
O
N
N
Y67
8
7
6
5
4
3
2
ppm
0.94
0.94
1.95
0.95
5.94
0.97
0.94
1.00
12.42
16.81
2.23
2.20
4.09
2.91
12.00

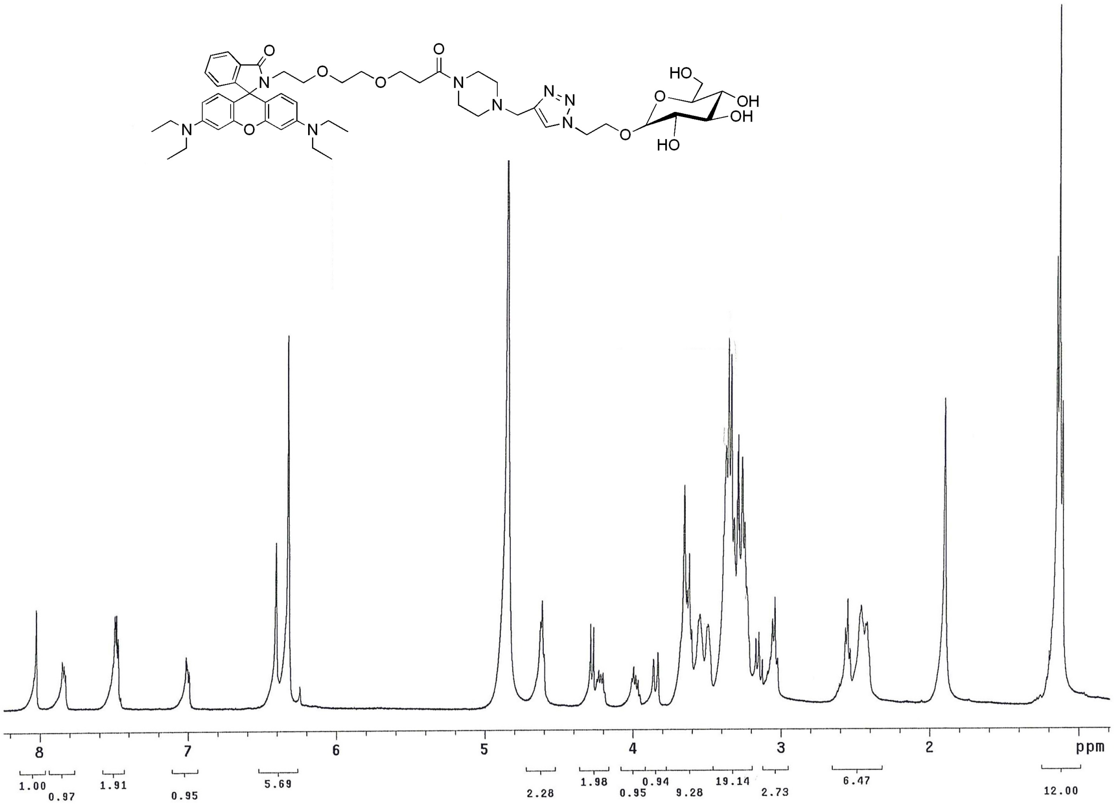

O
N
O
N
N
N
N
O
O
HO
OH
OH
HO
8
7
6
5
4
3
2
ppm
1.00
0.97
1.91
0.95
5.69
2.28
1.98
0.95
0.94
9.28
19.14
2.73
6.47
12.00

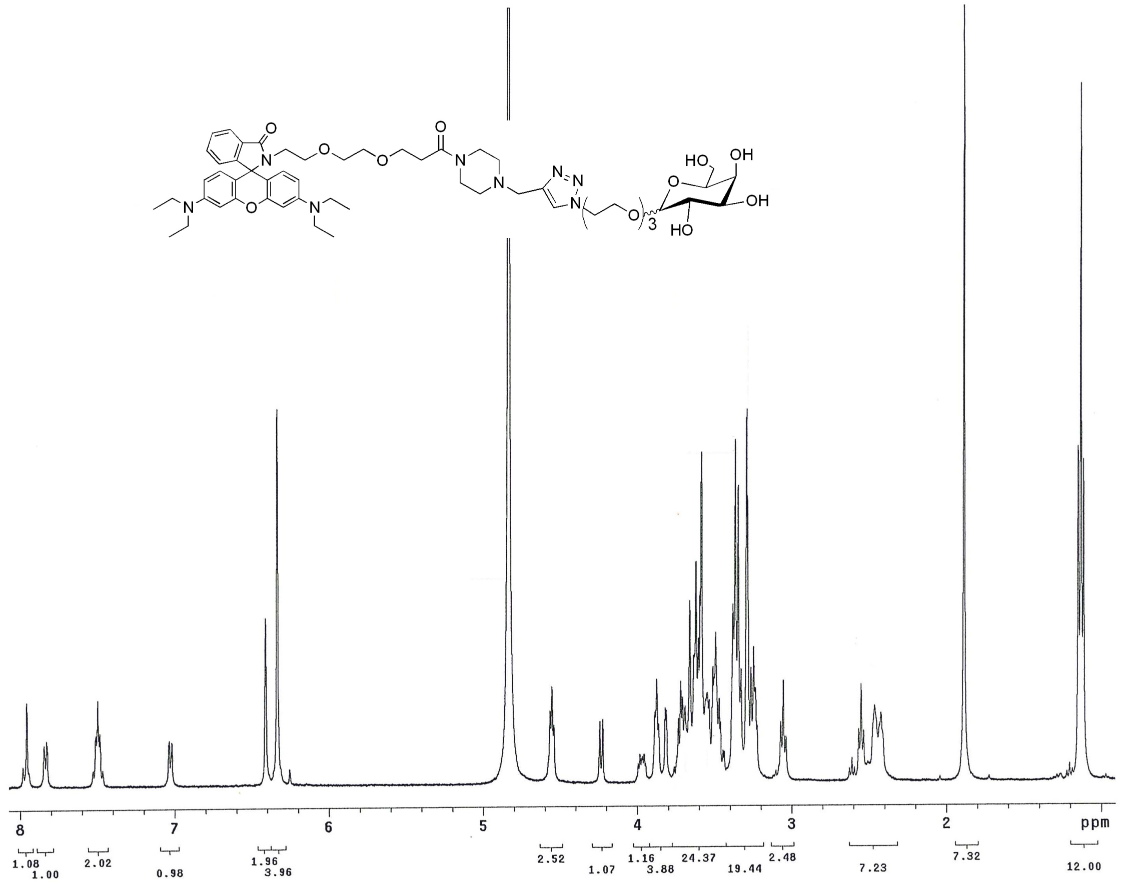
HO
OH
OH
HO
3
8
7
6
5
4
3
2
ppm
1.08
1.00
2.02
0.98
1.96
3.96
2.52
1.07
1.16
3.88
24.37
19.44
2.48
7.23
7.32
12.00

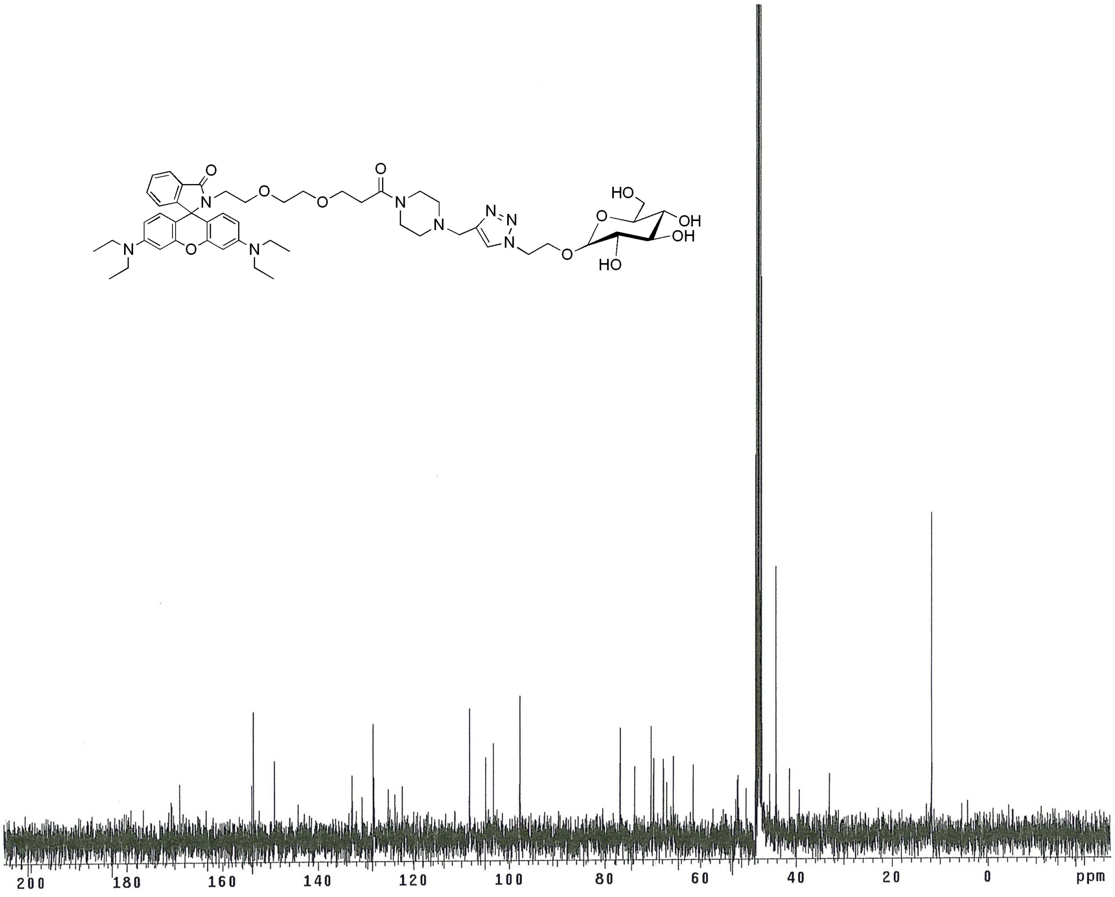
O
N
O
O
O
N
N
N
N
N
N
O
O
HO
OH
OH
HO
N
N
200
180
160
140
120
100
80
60
40
20
0
ppm

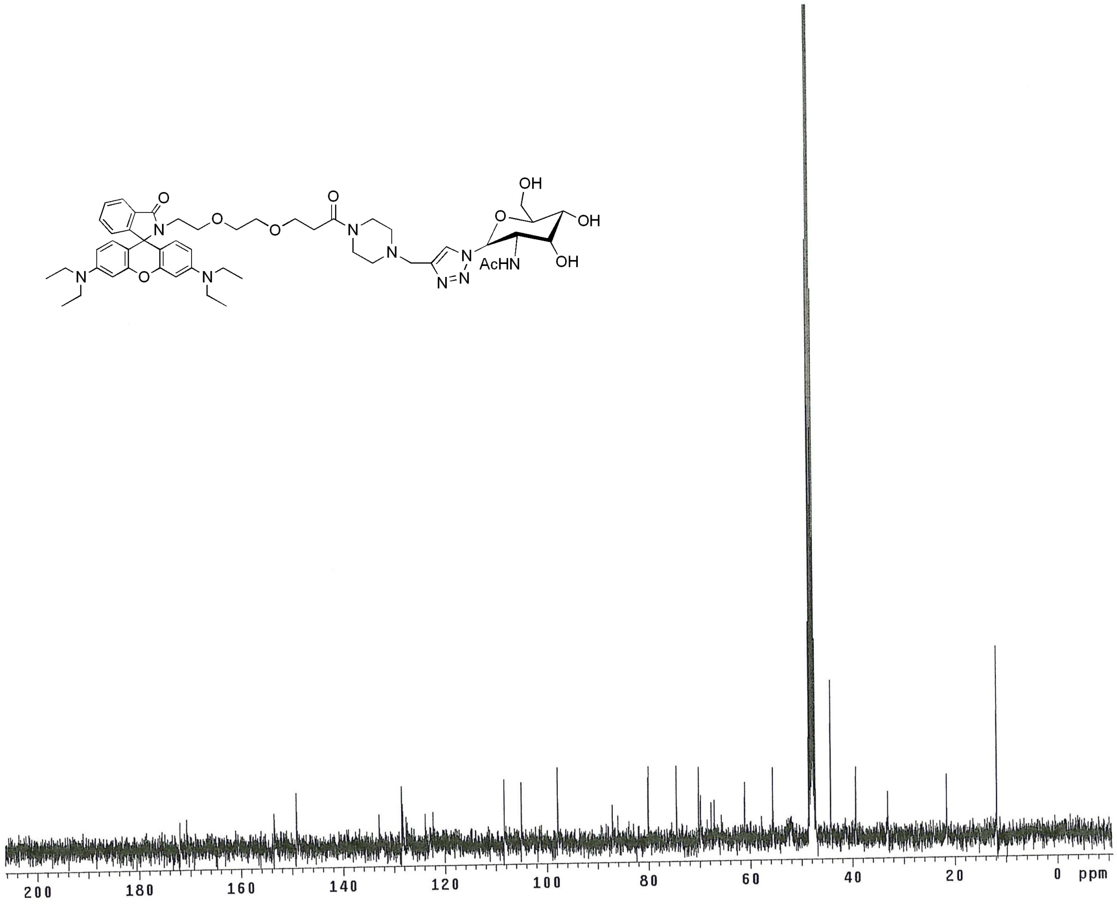
O
N
O
O
O
N
N
N
N
N
N
O
OH
OH
OH
AcHN
200
180
160
140
120
100
80
60
40
20
0 ppm

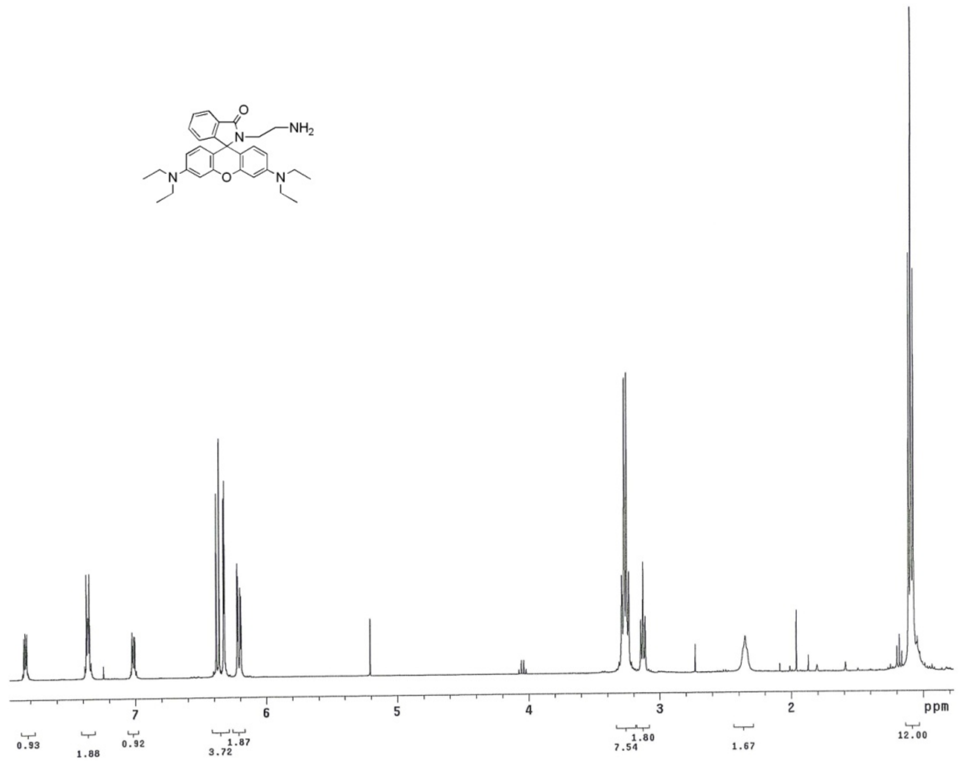
O
N
NH2
N
O
N
7
6
5
4
3
2
ppm
0.93
1.88
0.92
3.72
1.87
1.80
7.54
1.67
12.00

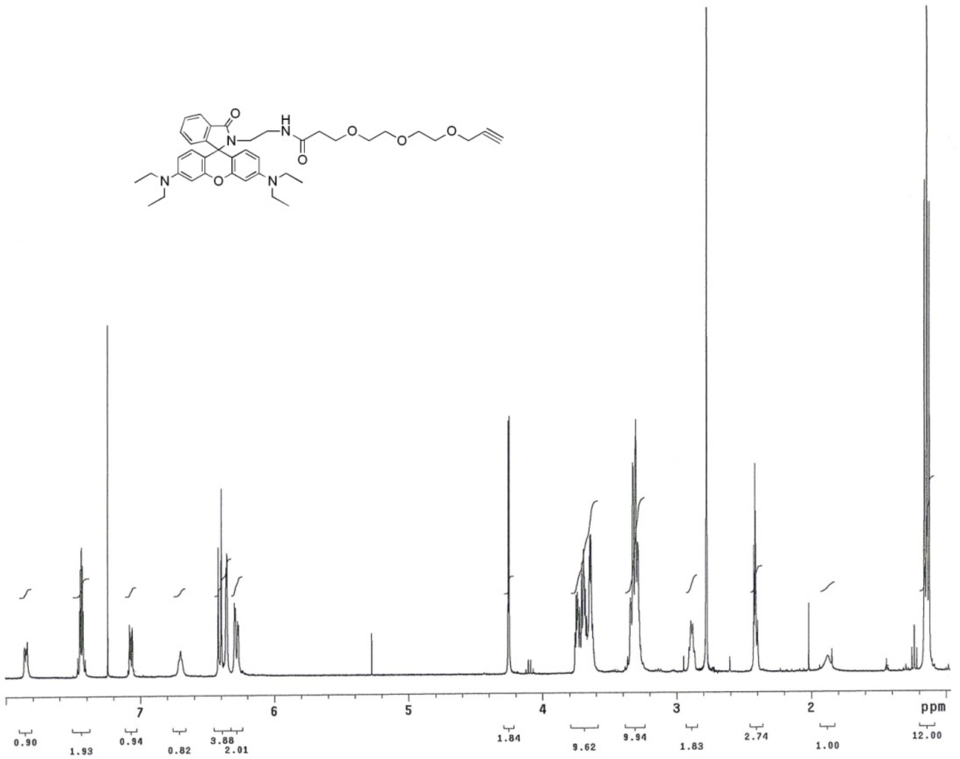
O
H
N
N
O
O
O
O
N
O
N
7
6
5
4
3
2
ppm
0.90
1.93
0.94
0.82
3.88
2.01
1.84
9.62
9.94
1.83
2.74
1.00
12.00

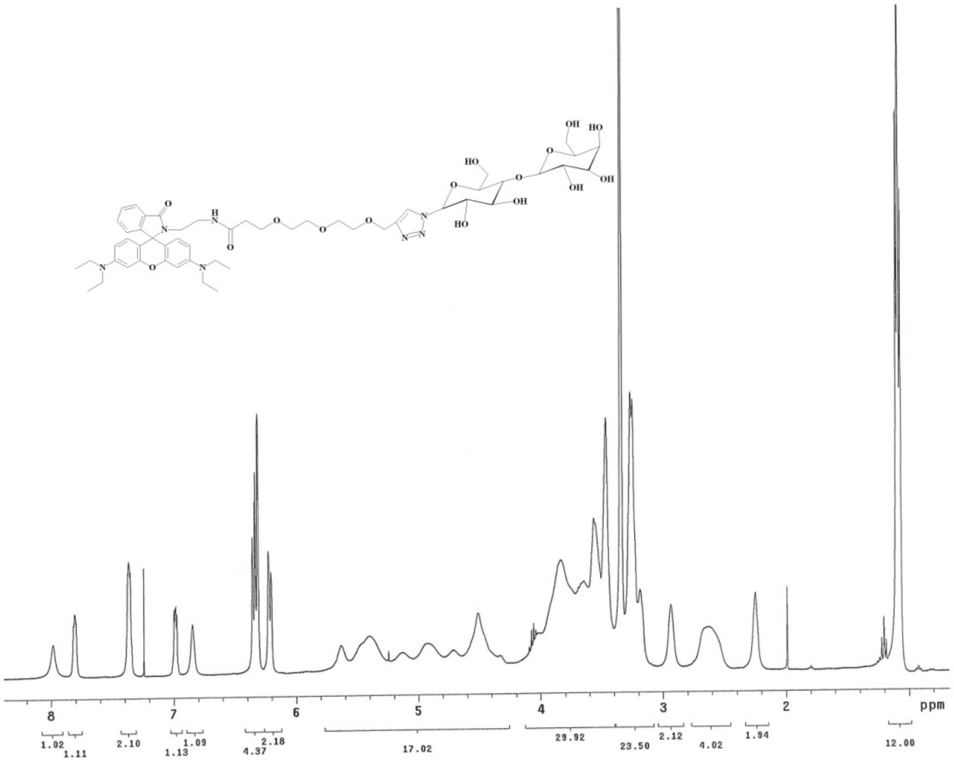
ppm
8
7
6
5
4
3
2
1.02
1.11
2.10
1.13
1.09
4.37
2.18
17.02
29.92
23.50
2.12
4.02
1.94
12.00
